# Estrogen-induced upregulation and 3′-UTR shortening of *CDC6*

**DOI:** 10.1093/nar/gks855

**Published:** 2012-09-12

**Authors:** Begum H. Akman, Tolga Can, A. Elif Erson-Bensan

**Affiliations:** ^1^Department of Biological Sciences and ^2^Department of Computer Engineering, METU (Middle East Technical University), Universiteler Mah, Dumlupınar Blv. No. 1, 06800 Çankaya, Ankara, Turkey

## Abstract

3′-Untranslated region (UTR) shortening of mRNAs via alternative polyadenylation (APA) has important ramifications for gene expression. By using proximal APA sites and switching to shorter 3′-UTRs, proliferating cells avoid miRNA-mediated repression. Such APA and 3′-UTR shortening events may explain the basis of some of the proto-oncogene activation cases observed in cancer cells. In this study, we investigated whether 17 β-estradiol (E2), a potent proliferation signal, induces APA and 3′-UTR shortening to activate proto-oncogenes in estrogen receptor positive (ER+) breast cancers. Our initial probe based screen of independent expression arrays suggested upregulation and 3′-UTR shortening of an essential regulator of DNA replication, *CDC6* (cell division cycle 6), upon E2 treatment. We further confirmed the E2- and ER-dependent upregulation and 3′UTR shortening of *CDC6*, which lead to increased CDC6 protein levels and higher BrdU incorporation. Consequently, miRNA binding predictions and dual luciferase assays suggested that 3′-UTR shortening of *CDC6* was a mechanism to avoid 3′-UTR-dependent negative regulations. Hence, we demonstrated *CDC6* APA induction by the proliferative effect of E2 in ER+ cells and provided new insights into the complex regulation of APA. E2-induced APA is likely to be an important but previously overlooked mechanism of E2-responsive gene expression.

## INTRODUCTION

mRNA 3′-UTRs (untranslated regions) significantly impact mRNA stability, localization and translational efficiency. As a major determinant of 3′-UTR length, polyadenylation starts with the endonucleolytic cleavage of mRNA at a site where polyadenylation factors bind, 10–35 residues downstream of the core consensus element (AAUAAA and possibly many other non-canonical variants). An adenosine (A) tail is subsequently added by the poly(A) polymerase (∼200 residues in higher eukaryotes) [reviewed in (1)]. Three types of polyadenylation events have been described [reviewed in (2)]. In Type I, only one polyadenylation signal exists and hence only one mRNA isoform is generated. In Type II, alternative polyadenylation (APA) signals exist in the terminal exon, generating mRNA isoforms of different 3′-UTR sizes that have identical coding sequences. In Type III, multiple polyadenylation signals exist in the upstream exons and introns, giving rise to alternatively spliced and alternatively polyadenylated mRNA isoforms. More than 50% of human genes are estimated to harbor Type III APA signals, possibly giving rise to different 3′-UTR-sized isoforms (3). Such APA sites seem to be utilized in diverse organisms such as plants and mammals, suggesting their fundamental functional significance in evolution (4,5).

Given that miRNAs are vital *trans *regulators of gene expression, recent studies showed that usage of proximal APA sites are highly relevant for miRNA-dependent regulation of mRNAs. For example, in response to an activation signal, the CD4+ T lymphocyte transcription profile was shown to switch to more proximal poly(A) sites to escape miRNA binding (6). Interestingly, such shorter 3′-UTR isoforms were also associated with the rapid proliferation of cells (6). In addition, switching to proximal APA sites and expressing shorter 3′-UTR isoforms, independent of proliferation signals, were shown in cancer cells as a way of escaping from miRNA control (7,8). Consequently, such global changes in 3′-UTR lengths were correlated with clinically distinct cancer subtypes (9). A more recent meta-analysis study evaluated large scale gene expression datasets and reported a correlation between 3′-UTR shortening and poor prognosis in breast and lung cancers (10). RNA sequencing in cancer cells further provided a general picture on APA-regulated transcript groups some of which were cell cycle, apoptosis and metabolism related (8). Another intriguing study showed APA for the β-actin transcript in mouse in a tissue-specific manner, which affected miRNA-mediated regulation (11).

It appears that the APA-mediated regulation of mRNA 3′-UTRs is a novel widespread mechanism of gene expression control which may be induced in response to proliferation signals, tissue specificity and tumorigenesis. Therefore, it is important to understand the APA-mediated regulation of potentially oncogenic mRNAs in response to proliferation signals in cancer cells as a rapid way of increasing translation by evading any 3′-UTR-dependent negative regulation including miRNAs. In this study, we examined whether 17 β-estradiol (E2), as a potent proliferation signal, induces APA to activate proto-oncogenes in estrogen receptor positive (ER+) breast cancer cells. To determine whether APA is induced due to E2, we developed a microarray analysis tool to screen for probe level differences in gene expression datasets. Using this *in silico* tool, we analysed three independent array datasets on E2-induced gene expression and found *CDC6* (*Cell Division Cycle 6*) as a common APA-regulated gene in response to E2. Transcriptional upregulation and APA for the *CDC6* transcript was confirmed and found to be both E2 and ER dependent. Our results showed that *CDC6* 3′-UTR short isoform was more efficiently translated compared with the longer isoform. Moreover, increased *CDC6* 3′-UTR short isoform levels indeed correlated with increased CDC6 protein levels and BrdU incorporation. In short, our results elucidate the use of APA for *CDC6* in response to E2 in breast cancer cells and will contribute to a more comprehensive understanding of E2 responsive gene expression changes in cancer cells. Such induced and/or deregulated APA events may also further explain some proto-oncogene activation cases where there are no causative genetic and/or epigenetic alterations identified.

## MATERIALS AND METHODS

### Probe level screen for APA transcripts

Three NCBI Gene Expression Omnibus (GEO) (12) experiment sets for E2 treatment in MCF7 cells, GSE11324 (13), GSE8597 (14) and GSE11791 (15), were selected. All three arrays were conducted on Affymetrix Human Genome U133 Plus 2.0 Arrays (HGU133Plus2, NCBI GEO Accession number: GPL570). CEL files were downloaded to use the non-normalized raw intensities of the probes. Affymetrix chip annotation files were used to map Unigene identifiers to probe set identifiers. To detect APA-dependent events in the 3′-UTRs, data sets were analyzed for probe level differences based on the positions of reported (16) poly(A) sites according to the UCSC Genome Browser Database (17). Proximal and distal probe sets were recorded based on the positions of multiple poly(A) sites that have been reported for 10 550 genes. With our analysis, we found that there were 4444 poly(A) sites that split 3067 probe sets into two subsets on the HG U133 Plus 2.0 chip. For each such case, the fold difference was computed between the proximal and distal probe sets of E2 treated and control datasets. All GEO series were analysed individually and their results were combined using a product approach to screen for transcripts that consistently had differently expressed split probe sets.

### Cell lines and treatments

MCF7, T47D, MDA-MB-231 cell lines were a kind gift from Dr. U.H. Tazebay (Bilkent University, Ankara). MCF7, MDA-MB-231 cells were grown in DMEM with Earle’s salts and 10% FBS, T47D cells were grown in RPMI, with 10% FBS and 0.1% non-essential amino acids. Media contained 1% penicillin/streptomycin (P/S). MDA-66 cell lines were a kind gift from Dr. U. H. Tazebay with permission from Dr. F. Gannon (18,19). MDA-66 cells were maintained in MDA-MB-231 medium with 0.4 mg/mL Hygromycin (Roche Applied Sciences). For MCF7 ERα silencing studies, MCF7_EV (Empty pSR vector transfected), MCF7_CO (Control shRNA transfected) (20), and MCF7_shERα (ER shRNA transfected clone) (18) cells were used. Stable cell lines were maintained with the addition of 0.25 mg/mL Geneticin (Roche Applied Sciences). Cell lines were grown as monolayers and were incubated at 37°C with 95% humidified air and 5% CO_2_.

For hormone induction experiments, cells were grown in phenol red-free medium supplemented with 10% charcoal-stripped FBS, 1% P/S and 1% l-glutamine for 48 h. MCF7 cells were either treated with 10 nM 17 β-Estradiol (E2) (Sigma-Aldrich) or ethanol (vehicle control) for 3 and 12 h, T47D cells were treated with 10 nM E2 (Sigma-Aldrich) or ethanol for 12 and 18 h (21–23). MDA-MB-231 cells were treated with 10 nM E2 (Sigma-Aldrich) or ethanol (vehicle control) for 3, 12 and 18 h (21–23). Cycloheximide (10 µg/mL; Sigma) (14) and 1 µM ICI 182,780 (Tocris Biosciences) (24) pre-treatments were done 1 h before E2 treatment.

### RNA isolation and cDNA synthesis

RNA isolation, quantification, cDNA synthesis and expression analysis were conducted according to MIQE Guidelines (25) and checklist (Supplementary Table S1). Total RNA was isolated using High Pure RNA Isolation Kit (Roche Applied Science). For DNase treatment, total RNA was incubated with DNase I (Roche Applied Science) at 37°C for 1 h followed by phenol-chloroform extraction (reaction conditions given in Supplementary Table S2). Lack of DNA contamination was confirmed by polymerase chain reaction (PCR) (Supplementary Figure S1A). RNA concentration and purity were determined by NanoDrop ND1000 (Thermo Scientific). cDNAs (20 µl) were synthesized using the RevertAid First Strand cDNA Synthesis Kit (Fermentas) using 2 μg total RNA and oligo dT primers (reaction conditions given in Supplementary Table S3).

### Expression analysis

For reverse transcriptase quantitative PCR (RT-qPCR), SYBR Green Mastermix (Roche Applied Science) was used using the Rotor Gene 6000 (Corbett, Qiagen) cycler. Twenty microliters of reactions were performed with 300 nM of specific primer pairs. The *CDC6* (NM_001254) short and long 3′-UTR isoforms were amplified using the following primer sets; *CDC6* 3′-UTR*-*short (product size size: 185 bp) *CDC6*_F:5′-TTCAGCTGGCATTTAGAGAGC-3′, *CDC6*_R1: 5′-AAGGGTCTACCTGGTCACTTTT-3′, *CDC6* 3′-UTR-long (product size size: 349 bp) *CDC6*_F:5′-TTCAGCTGGCATTTAGAGAGC-3′, *CDC6*_R2: 5′-CGCCTCAAAAACAACAACAA-3′. The fold change for the isoforms was normalized against the reference gene; *SDHA *(NM_004168) (26) amplified with the following primer sets: *SDHA *(product size: 86 bp) *SDHA*_F: 5′-TGGGAACAAGAGGGCATCTG-3′ and *SDHA*_R: 5′- CCACCACTGCATCAATTCATG-3′. Reference gene, *SDHA,* expression is consistent across breast cancer cell lines (26) and did not change in response to E2 treatment (data not shown). For all three reactions, following conditions were used: incubation at 94°C for 10 min, 40 cycles of 94°C for 15 s, 56°C for 30 s and 72°C for 30 s (Supplementary Table S4, Supplementary Figures S1–3). *E2F1, E2F2, CSTF2, CSTF3* and *CPSF2* primer sequences were taken from the literature (27,28).

For the relative quantification, the reaction efficiency incorporated ΔΔCq formula was used (29). Three independent biological replicates with three technical replicas per experiment were used for each PCR. One-way ANOVA with Tukey’s multiple comparison post test was performed using GraphPad Prism (California, USA). *TFF1* (aka pS2) expression was determined by RT-PCR as a positive control for the E2 treatment (18).

### Rapid amplification of cDNA ends

Rapid amplification of cDNA ends (RACE)-specific cDNA synthesis was performed using the 3′ RACE Kit (Roche) with 2 μg total RNA (DNase treated) from E2-treated MCF7 cells using the oligo dT-anchor primer (5′-GACCACGCGTAT CGATGTCGACTTTTTTTTTTTTTTTTV-3′).

For PCR, following Gene Specific Primers were designed and used:

3′RACE_1: 5′-GCTCTTGGAAGCCAGGGGCATTTTA-3′,

3′RACE_2: 5′-CCACCCGAAAGTATTCAGCTGGCATTTA-3′.

A reverse primer for the anchor sequence was used (Anchor-R: 5′-GACCACGCGTATC GATGTCGAC-3′). First round 3′RACE PCR was done using the 3′RACE_1 and Anchor-R primers with the following PCR conditions; 94°C for 10 min, 20 cycles of 94°C for 30 s, 64°C for 30 s, 72°C for 30 s. Nested 3′RACE PCR was performed using the 3′RACE_2 and Anchor-R primers with the 1/10 diluted PCR product as template with the following PCR conditions; incubation at 94°C for 10 min, 35 cycles of 94°C for 30 s, 64°C for 30 s, 72°C for 30 s. Following gel extraction of correct size bands with High Pure Gel Extraction Kit (Roche), another round of 3′RACE PCR was performed with 2 ng of PCR product as the template using 3′RACE_2 and Anchor-R primers with the following PCR conditions; incubation at 94°C for 10 min, 35 cycles of 94°C for 30 s, 64°C for 30 s, 72°C for 30 s.

### Protein isolation and western blotting

Cells were washed with phosphate-buffered saline (PBS) and nuclear extraction buffer (20 mM hydroxyethyl piperazineethanesulfonic acid (HEPES), 10 mM KCl, 0.1 mM ethylenediaminetetraacetic acid, 1 mM Dithiothreitol (DTT), 1.5 mM MgCl_2_, 0.5 mM NaCl, 10% NP-40, 25% glycerol). A tablet cocktail of protease inhibitors (Roche) dissolved in 10 mL dH_2_O was used to isolate nuclear proteins as described (30). BCA Protein Assay Kit (Pierce) was used for lysate quantification. Nuclear extracts (50 μg) were denatured in 6X Laemmli buffer (12% sodium dodecyl sulphate, 30% 2-mercaptoethanol, 60% glycerol, 0.012% bromophenol blue, 0.375 M Tris) at 100°C for 5 min and were separated on a 8% polyacrylamide gel and transferred onto a nitrocellulose membrane (Bio-Rad). The membranes were blocked in 5% non-fat milk in TBS-T (Tris Buffer Saline-Tween, 20 mM Tris, 137 mM NaCl, pH: 7.6, 0.1% Tween 20) and were incubated overnight with the appropriate primary antibodies: monoclonal anti-CDC6 rabbit antibody (1:1000 dilution; Cell Signaling); followed by a 1 h incubation with the HRP-conjugated secondary anti-rabbit antibody (1:2000 dilution, Cell Signaling). Proteins were visualized using an enhanced chemiluminescence kit (ECL Plus; Pierce) according to the manufacturer’s instructions. For nuclear protein loading control, Histone H3 (FL-136) antibody, a gift from Dr. S. Banerjee (METU, Ankara) (Santa Cruz Biotechnology), was used. Five percentage non-fat milk in PBS-T was used for blocking. Primary antibody was 1:500 diluted and secondary anti-rabbit (Santa Cruz Biotechnology) antibody was 1:2000 diluted. ERα (F-10) antibody (sc-8002, Santa Cruz Biotechnology) was used for detecting ERα protein. Five percentage of non-fat milk in TBS-T was used for blocking. Primary antibody was 1:200 diluted and secondary anti-mouse (Santa Cruz Biotechnology) antibody was 1:2000 diluted. β-actin antibody (sc-47778, Santa Cruz Biotechnology) was used for protein loading control. For blocking, 5% BSA in TBS-T was used. Primary antibody was 1:1000 diluted and secondary anti-mouse antibody was 1:2000 diluted.

### BrdU incorporation

1 × 10^4^ MCF7, T47D and MDA-66 cells were seeded and grown in black 96-well plates (Canberra Packard) in 100 μl of DMEM-10% FBS or RPMI-10% FBS, respectively. A day later, media were changed to 10% charcoal stripped FBS-medium and cells were incubated for 48 h. BrdU incorporation together with E2 treatment was pursued for 0, 3, 12, 24 h for MCF7; 0, 12, 18, and 24 h for T47D; 0, 3, 12, 18 h for MDA-66. BrdU incorporation was detected according to the protocol from Cell Proliferation ELISA, BrdU assay kit (Roche Applied Science) using the Turner Biosystems Luminometer. Readings are presented as relative light units per second (RLU/sec) at respective time points. Two independent assays were performed with five replicates per experiment.

### microRNA target sites on mRNAs

TargetScan (http://www.targetscan.org/), PicTar (http://pictar.mdc-berlin.de/) and FindTar3 (http://bio.sz.tsinghua.edu.cn/) were used to predict possible miRNA–mRNA interactions.

### Dual luciferase analysis

Short (341 bp) and long (915 bp) 3′-UTR fragments were PCR amplified with cloning primers using a high fidelity polymerase (Fermentas *Pfu *polymerase) into pMIR-Report (pMIR) plasmid (Ambion). SacI and HindIII recognition site harboring cloning primers were as follows: (restriction sites are in bold letters and recognition aiding sites are italicized).

CDC6_pMIR_F: 5′-*C***GAGCTC***G*ATTCTTCTCTTACACCCCAC-3′

CDC6_pMIR_R1: 5′-*CCC***AAGCTT***GGG*AAAATACCCACTCATGTTTGAG-3′

CDC6_pMIR_R2: 5′-*CCC***AAGCTT***GGG*AACTTGAAAATAAATATATTC-3′

After sequence confirmation, MCF7 cells were co-transfected with pMIR (Firefly luciferase) (375 ng) and phRL-TK (Renilla Luciferase) (125 ng) in 24-well plates using 0.75 μL Fugene-HD (Roche). Twenty-four hours after transfection, cells were collected and dual luciferase activities were measured using the Modulus Microplate Luminometer (Turner Biosystems). The luminescence intensity of Firefly luciferase (pMIR) was normalized to that of Renilla luciferase (phRL-TK). Experiments were repeated three independent times with four replicates per experiment. For E2 treatments, MCF7 cells were kept in phenol red-free MEM supplemented with 10% charcoal-stripped FBS, 1% P/S and 1% l-glutamine for 48 h. MCF7 cells were then co-transfected with pMIR and phRL-TK constructs as described earlier. Twelve hours after transfection, cells were treated with 10 nM E2 for 12 h. E2 responsive TFF1-ERE in pGL3 (18) was used as an E2 treatment control. Experiments were repeated two independent times with three replicates per experiment.

## RESULTS

**Figure 1.**
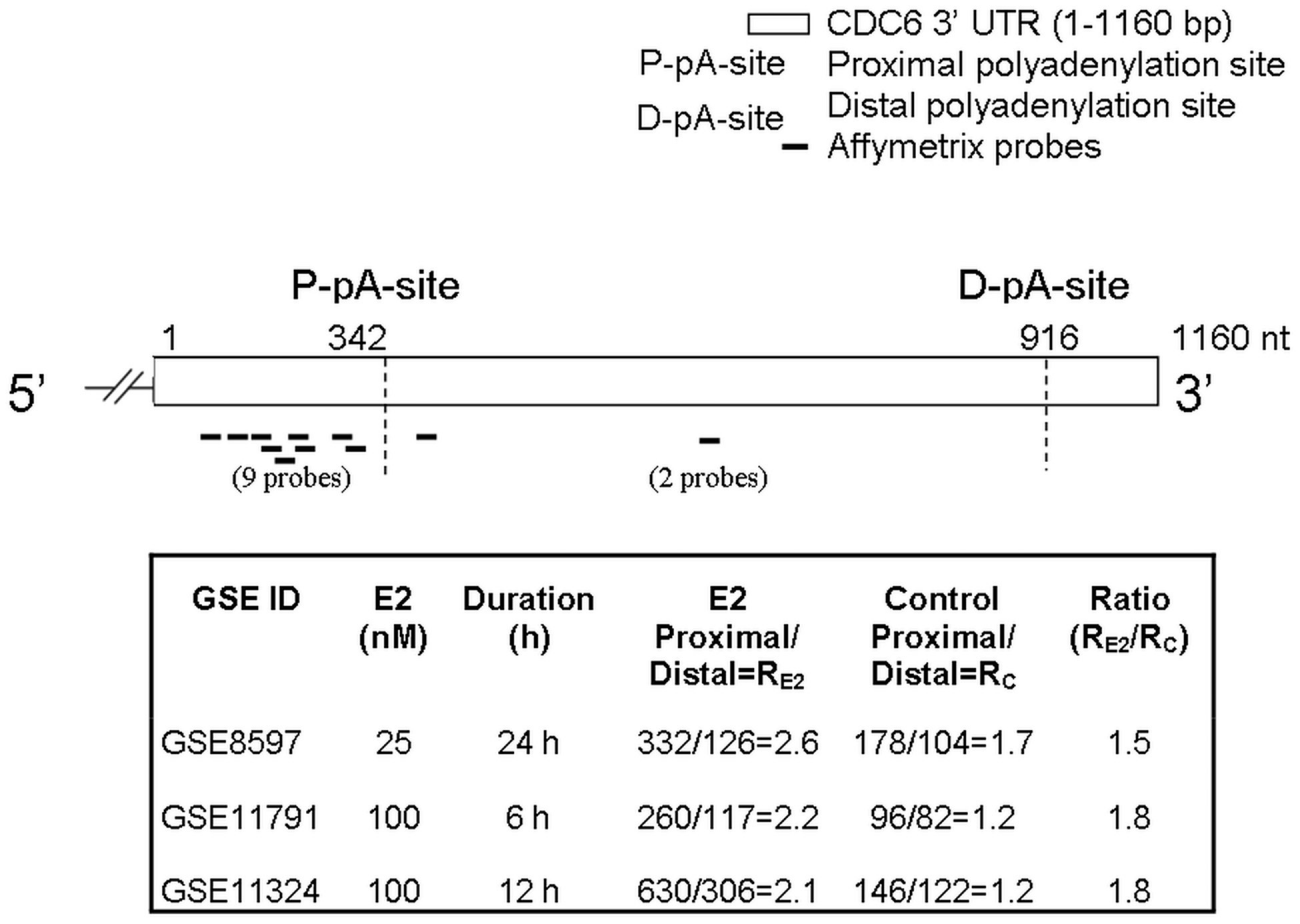
*CDC6 *3′-UTR (1160 nucleotides) and the positions of the probes are shown. Nine of the probes matched to the site upstream of the proximal poly(A) (P-pA) site and two of the probes matched to the region between P-pA and distal poly(A) (D-pA) site. P-pA and D-pA are the reported poly(A) sites (16). Three independent gene expression datasets for E2 treatment in MCF7 cells (GSE8597, GSE11791, GSE11324) were analysed at the probe level. The mean of nine proximal and two distal probe set signals were collected. The proximal to distal signal ratio in E2-treated (R_E2_) MCF7 cells increased at least 1.5× fold compared with the ratios (*R*_C_) of the control cells.

Proximal APA, in response to proliferative signals, causes 3′-UTR shortening and contributes to the rapid proliferation of cells (6). Therefore, we investigated whether E2, a potent proliferation signal, induces APA and 3′-UTR shortening as a means to activate proto-oncogenes in ER+ breast cancer cells. To identify transcripts regulated by APA, we developed a gene expression array analysis tool to detect probe level differences. Transcript-specific individual probes were grouped into proximal and distal sets based on the positions of the reported 3′-UTR poly(A) sites (16). The average proximal to distal probe signal intensity values in E2-treated cells were compared with that of untreated (control) cells. Using this approach, we were able to detect proximal/distal probe set ratio differences caused by APA in response to E2. Three independent gene expression datasets (described in the Materials and Methods section) generated with E2 or ethanol (vehicle control) treated MCF7 cells were analysed. *CDC6*, regulator of the DNA pre-replication complex was identified as a common candidate in all three datasets due to its increased proximal to distal probe signal ratio in response to E2 (Figure 1). Next, *CDC6* was experimentally investigated for possible APA and generation of a shorter 3′-UTR isoform in response to E2 to confirm the *in silico* results.

**Figure 2.**
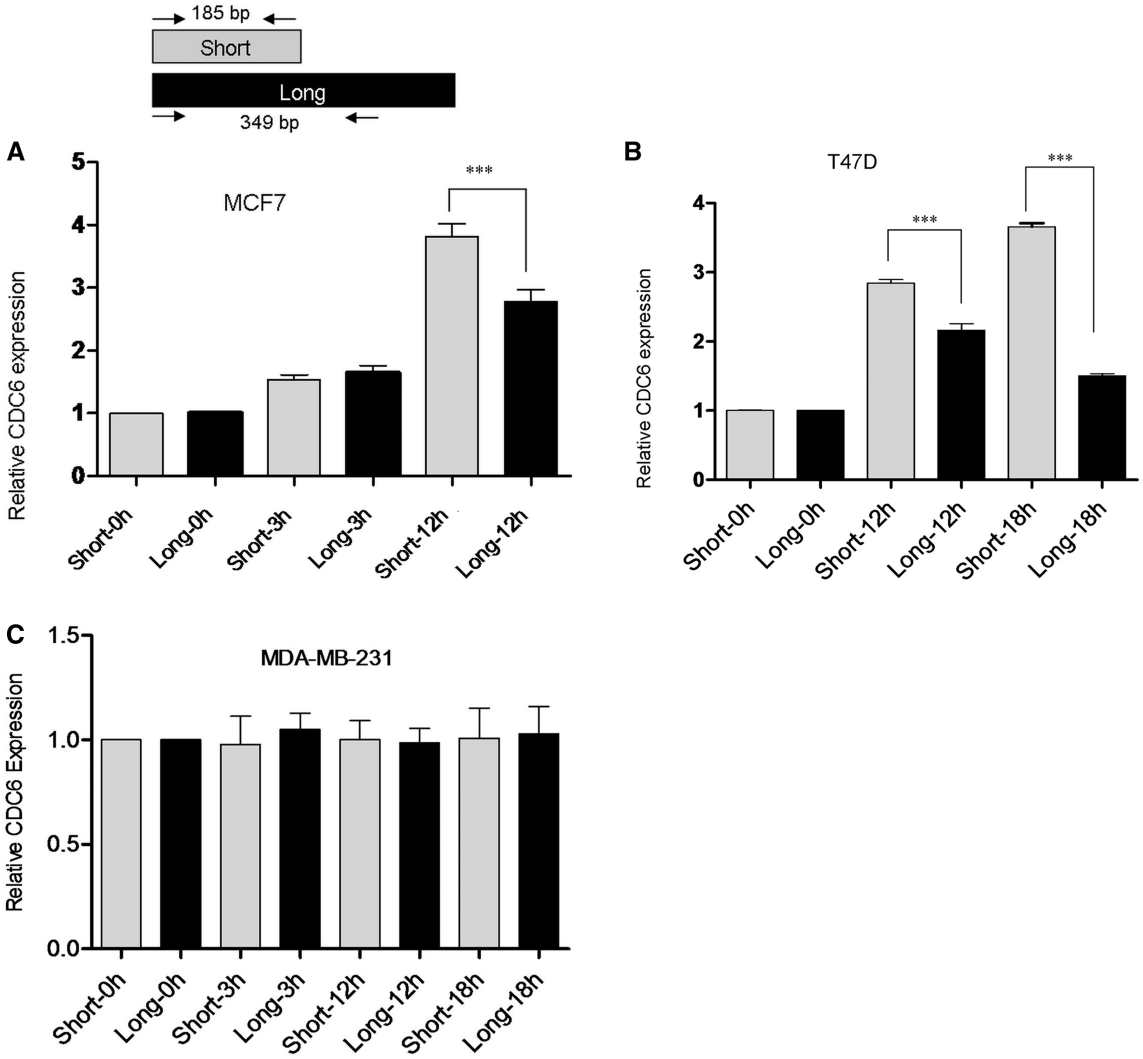
Relative quantification of *CDC6* 3′-UTR short and long isoforms in E2-treated and control cells. (**A**) MCF7 cells were treated with 10 nM E2 for 3 and 12 h. (**B**) T47D cells were treated with 10 nM E2 for 12 and 18 h. (**C**) MDA-MB-231 cells were treated with 10 nM E2 for 3, 12 and 18 h. The fold change for the isoforms was normalized against the reference gene, *SDHA. *Quantification was done using the reaction efficiency correction and ΔΔCq method (29). The baseline for the short and long isoforms in untreated samples was set to 1. ***Indicates significant difference between short and long isoforms’ expression, *P* < 0.001 (one-way ANOVA followed by Tukey’s multiple comparison test).

**Figure 3.**
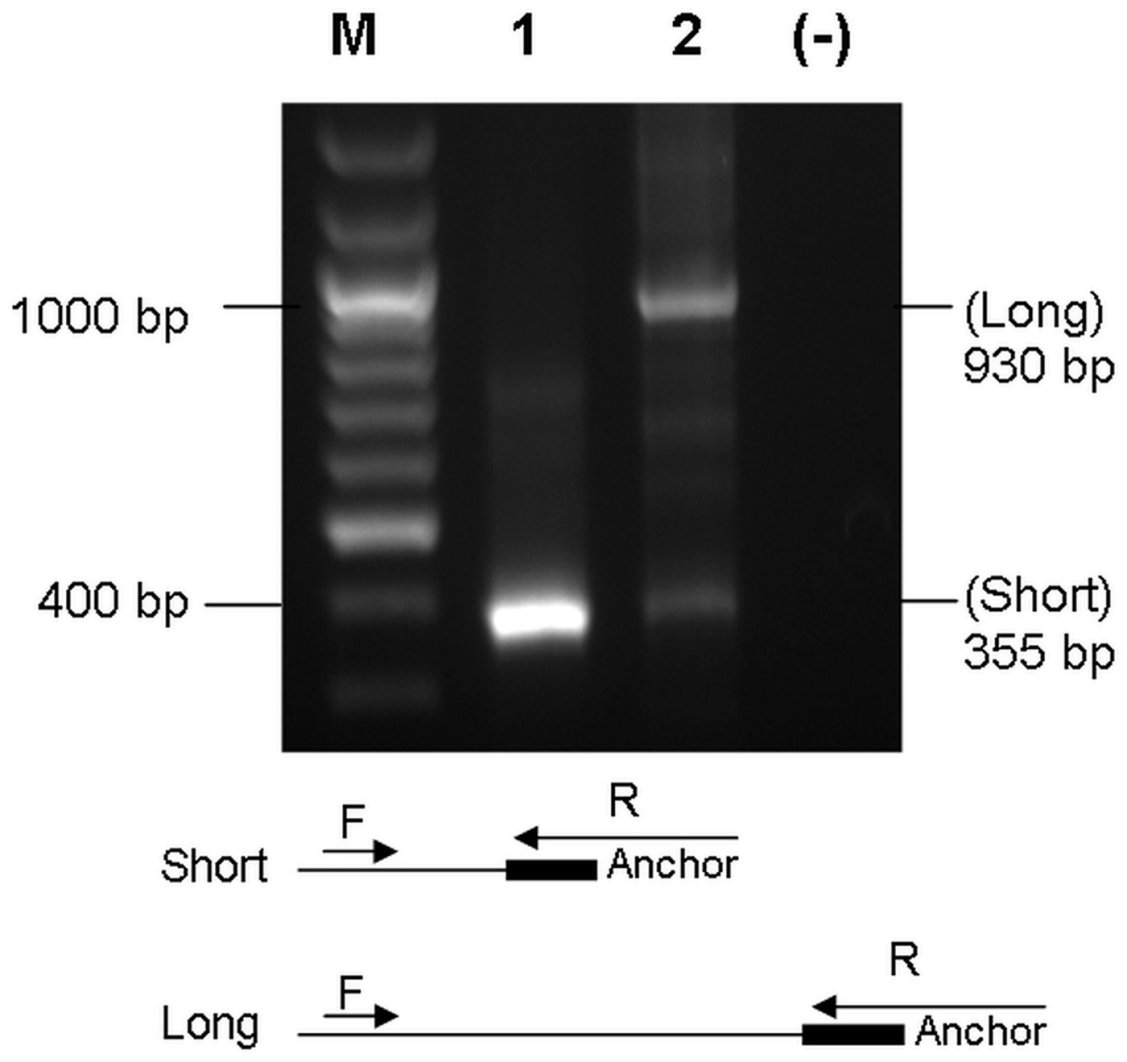
3′ RACE confirmed the existence of the short isoform. cDNA was synthesized using E2-treated MCF7 total RNA with anchor oligo-dT using the 3′ RACE Kit (Roche Applied Sciences). *CDC6* gene-specific forward (F) and anchor-specific reverse (R) primers amplified the expected 355 bp (lane 1, 2) and 930 bp (lane 2) products of the short and the long isoforms after nested 3′ RACE. Three hundred fifty-five base pairs of PCR product in lane 2 corresponding to the short isoform was gel purified and used as a PCR template (lane 1) with the *CDC6* forward and R anchor primers. Sequencing of this product (lane 1) verified the presence of the poly(A) tail. M: DNA ladder, (−): No template reaction.

MCF7 cells (ER+) were treated with either 10 nM E2 or ethanol (vehicle control) for 3 and 12 h and RNA was isolated. The effect of E2 treatment was confirmed with the upregulated expression of *TFF1, *a known E2 target gene (Supplementary Figure S4). In accordance with the *in silico* analysis, *CDC6* 3′ short isoform expression was found to increase approximately 38% more than the longer 3′-UTR isoform in response to E2 (Figure 2A). To test whether these findings were cell line specific responses, a second ER+ breast cancer cell line, T47D, was treated with E2 and expression of short and long 3′-UTR isoforms were quantified. The effect of E2 treatment was again confirmed with the upregulation of known E2-induced *TFF1* expression detected by RT-PCR (Supplementary Figure S4). Similar to E2-treated MCF7 cells; a robust increase of the short 3′-UTR isoform of *CDC6* was detected only in E2-treated T47D cells (Figure 2B). In contrast to the upregulation and 3′ shortening of *CDC6* in response to E2 in ER+ cells (Figure 2A and B), when an ER negative cell line (MDA-MB-231) was treated with E2, there was neither an upregulation nor 3′-UTR shortening of *CDC6* (Figure 2C). In support of our findings, MDA-MB-231 growth rate is known to be unaffected by E2 (21–23). For isoform expression analyses, RT-qPCR efficiency differences of the short and long isoforms were incorporated into the relative expression quantification calculation (29). However, because the short 3′-UTR primers also amplified the long 3′-UTR isoform, a further validation was needed to prove the existence of the short 3′-UTR. Nested 3′ RACE using a gene-specific forward primer and an oligo dT-anchor reverse primer produced the expected size for the short and the long 3′-UTR isoforms in E2-treated MCF7 cells (Figure 3). Sequencing the PCR product for the short 3′-UTR verified the presence of the proximal poly(A) site and the poly(A) tail (Supplementary Figure S5), which further confirmed the existence of the short transcript.

**Figure 4.**
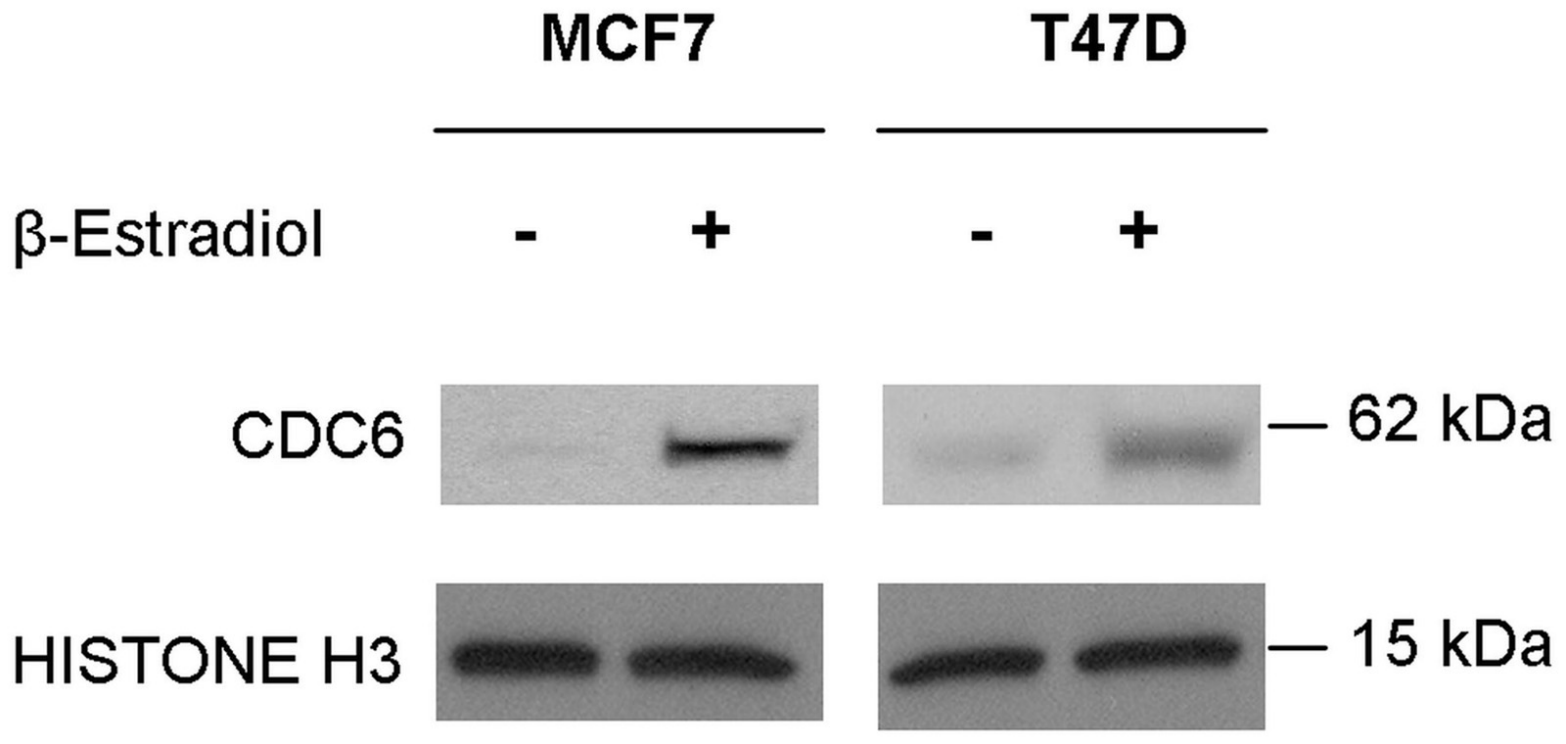
E2 treatment caused increase of the CDC6 protein levels. MCF7 and T47D nuclear lysates were collected followed by 10 nM E2 treatment for 12 and 18 h, respectively. Histone H3 antibody was used as a nuclear protein loading control.

Given the role of CDC6 as the initiator of pre-replication complex formation (31,32), we then investigated whether increased *CDC6* transcript levels indeed correlated with increased protein levels and DNA replication. Western blot analysis showed that CDC6 protein levels increased in response to E2 treatment in both cell lines (Figure 4). Next, asynchronous MCF7 and T47D cells were either treated with E2 or ethanol along with BrdU. The incorporation of BrdU into DNA in E2-treated cells compared with controls significantly increased at around the same time *CDC6* 3′-UTR short isoform and protein levels increased in E2-treated MCF7 and T47D cells (Supplementary Figure S6).

**Figure 5.**
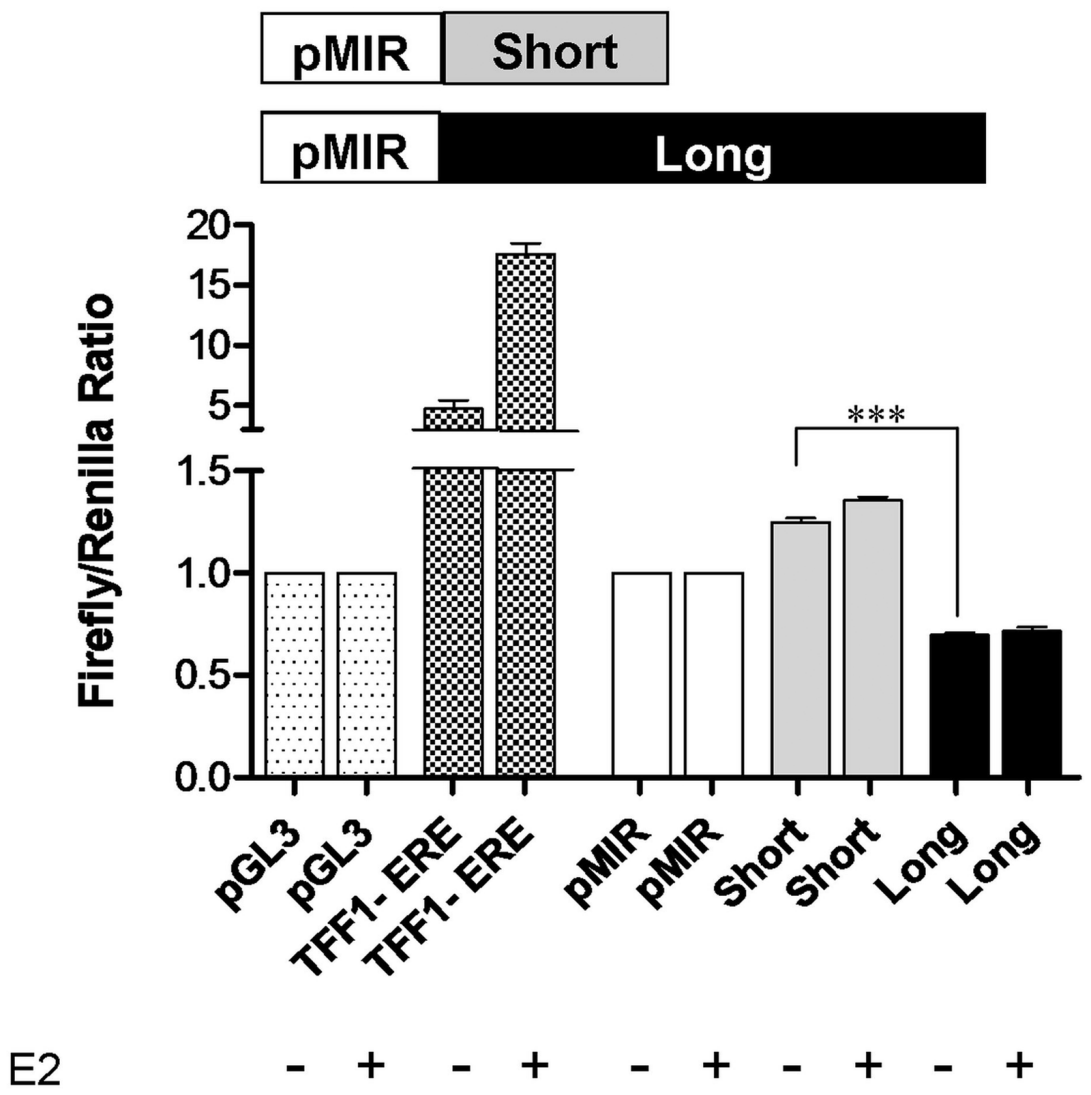
Dual luciferase reporter assay. *CDC6 *short and long 3′-UTRs were cloned into the 3′-UTR of Firefly luciferase gene in pMIR. pMIR–short, pMIR–long and pMIR–empty vectors were co-transfected with phRL–TK into MCF7 cells. MCF7 cells were kept in phenol red-free medium supplemented with 10% charcoal-stripped FBS for 48 h. Twelve hours post-transfection, E2 was applied to a final concentration of 10 nM for 12 h. E2 responsive *TFF1*-promoter construct was used as a positive control for E2 treatment. pGL3 is the empty vector, TFF1-ERE (in pGL3) harbors the promoter and E2-responsive regions of the *TFF1* gene. Dual-luciferase assay was performed 24 h after transfection. Firefly/Renilla luciferase read-outs from the constructs were normalized to that of empty pMIR, which was set to 1. ***Indicates *P* < 0.001 (one-way ANOVA followed by Tukey’s multiple comparison test).

We then investigated whether the E2 induced increase of the *CDC6* 3′-UTR short versus the long isoform was a mechanism that would allow for a more robust increase in protein levels by evading miRNA binding. First, we used miRNA target prediction tools to detect whether there were any *CDC6* 3′-UTR long isoform specific miRNAs. Three different miRNA target prediction programs (PITA, FindTar3 and TargetScan) predicted more than 60 miRNAs binding to the *CDC6* 3′-UTR, but interestingly, 87% of all predictions were localized to the region between the proximal poly(A) site (P-pA-Site) and the distal poly(A) site (D-pA-Site), a region which signifies the long 3′-UTR (Table 1). More intriguingly, the intersection of all three prediction sets gave rise to only four common miRNAs (miR-25, miR-541, miR-92a and miR-92b), which were predicted to bind only to the long 3′-UTR. No common miRNA predictions were present for the short 3′-UTR (Table 1). To test whether the *CDC6* long 3′-UTR actually harbored miRNA binding sites which were not present in the short 3′-UTR, we cloned the short and long 3′-UTR sequences into the 3′-UTR of the Firefly Luciferase gene of a reporter plasmid (pMIR). Short and long 3′-UTR constructs in pMIR along with phRL-TK (Renilla Luciferase), for normalization of transfection, were then transfected into MCF7 cells. Of interest, the short 3′-UTR construct had a significantly higher (85%) luciferase activity compared with the long 3′-UTR construct (Figure 5). These results suggested that the short 3′-UTR transcript was more efficiently translated which may explain how CDC6 protein levels increased in response to E2.

**Table 1.** miRNA target prediction programs (PITA, FindTar3 and TargetScan) predicted 67 miRNAs to bind to *CDC6* 3′-UTR

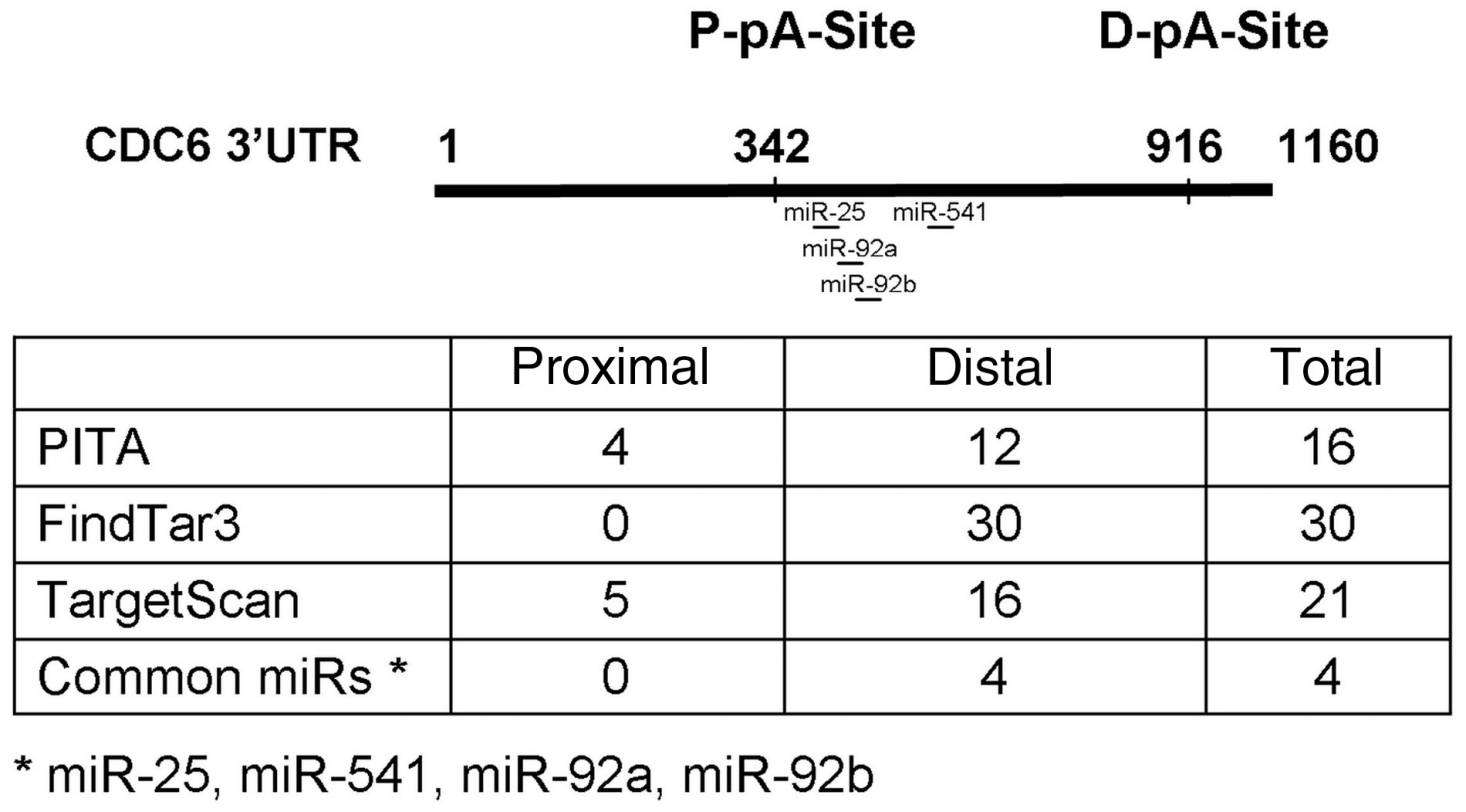

Fifty-eight of the predictions were only for the long isoform-specific region, marked by the proximal polyA site (P-pA-Site) and the distal polyA site (D-pA-Site). The intersection of all three prediction sets gave rise to four common miRNAs (miR-25, miR-541, miR-92a and miR-92b) which were predicted to bind only to the long 3′-UTR. No common miRNA predictions were present only for the short 3′-UTR.

Next, we investigated the mechanism of how *CDC6* 3′-UTR short isoform increased more than the longer isoform in E2-treated cells. For that, we considered two possibilities; (i) post-transcriptional regulation by E2-regulated miRNAs to explain increased short isoform relative to long isoform in response to E2 or (ii) E2 induced the transcriptional upregulation and APA of *CDC6* to generate a shorter 3′-isoform.

First, we examined the possibility of E2 regulated miRNAs to explain increased short isoform levels. Hence, short and long 3′-UTR harboring reporter constructs along with the phRL-TK (Renilla Luciferase) vector were transfected into ER+ cells that were treated with E2 or ethanol. Although the luciferase activity difference of the short versus the long 3′-UTR constructs was still evident, the difference was E2 independent. Interestingly, there was also no change in the luciferase activity of the short or the long 3′-UTR construct in response to E2 treatment compared with respective untreated short or long isoform constructs (Figure 5). The effect of E2 treatment was verified with the approximately 4-fold upregulation of luciferase expressed from the E2 responsive promoter region of *TFF1* gene as a positive control (Figure 5). Lack of any E2 induced change in luciferase activity for isoforms suggested that E2 did not have a post-transcriptional effect on the isoforms such as binding of E2 up-regulated miRNAs and/or increased degradation of the long isoform in response to E2. In essence, luciferase reporter assays in E2-treated cells indicated that E2 effect on CDC6 protein increase was more likely to be transcriptional, i.e. through APA and switching to a shorter 3′-UTR, rather than post-transcriptional mechanisms.

**Figure 6.**
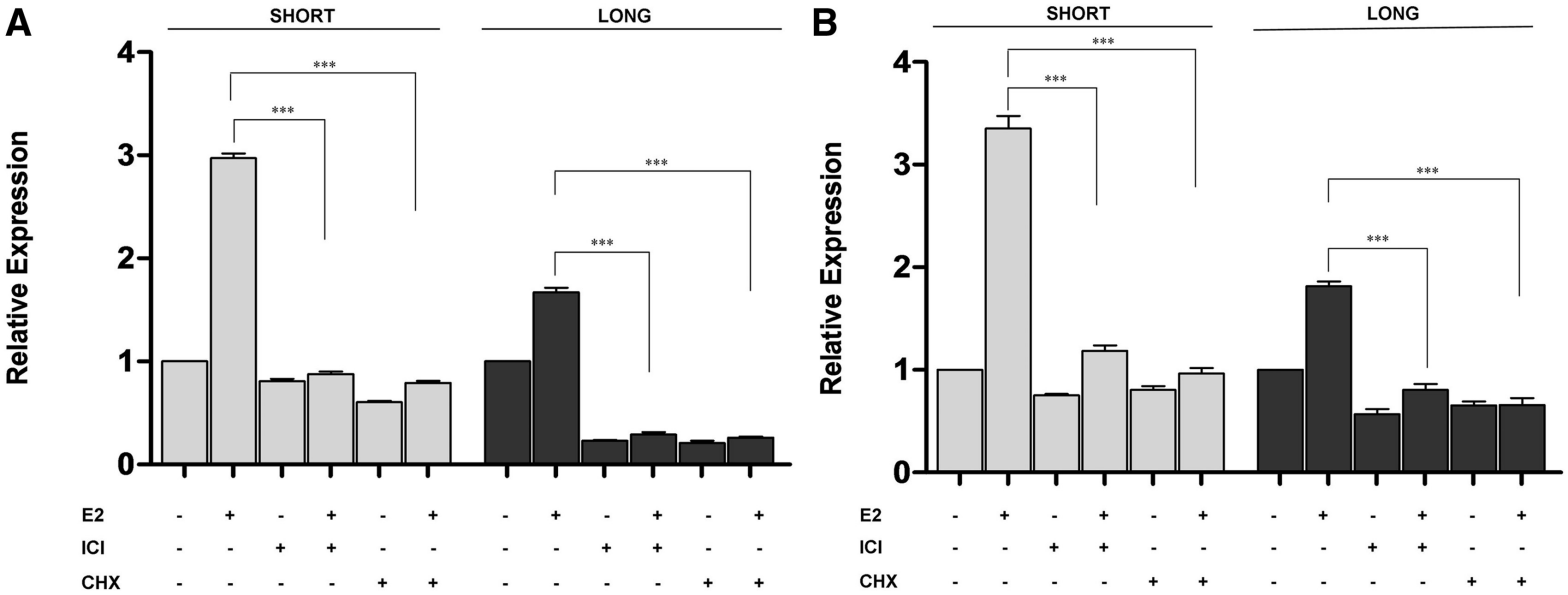
E2-induced *CDC6* expression requires ER and *de novo* protein synthesis. (**A**) MCF7 and (**B**) T47D cells were grown in phenol red-free medium supplemented with 10% dextran-coated-charcoal stripped FBS, pre-treated with 1 µM ICI or with 10 µg/mL CHX (Cycloheximide) for 1 h, then with 10 nM E2 for 12 h. Relative expression of *CDC6 *short (gray bars) and long (black bars) isoforms was determined by RT-qPCR. The baseline for the control-treated samples was set to 1. ***Indicates statistical significance (*P* < 0.001).

**Figure 7.**
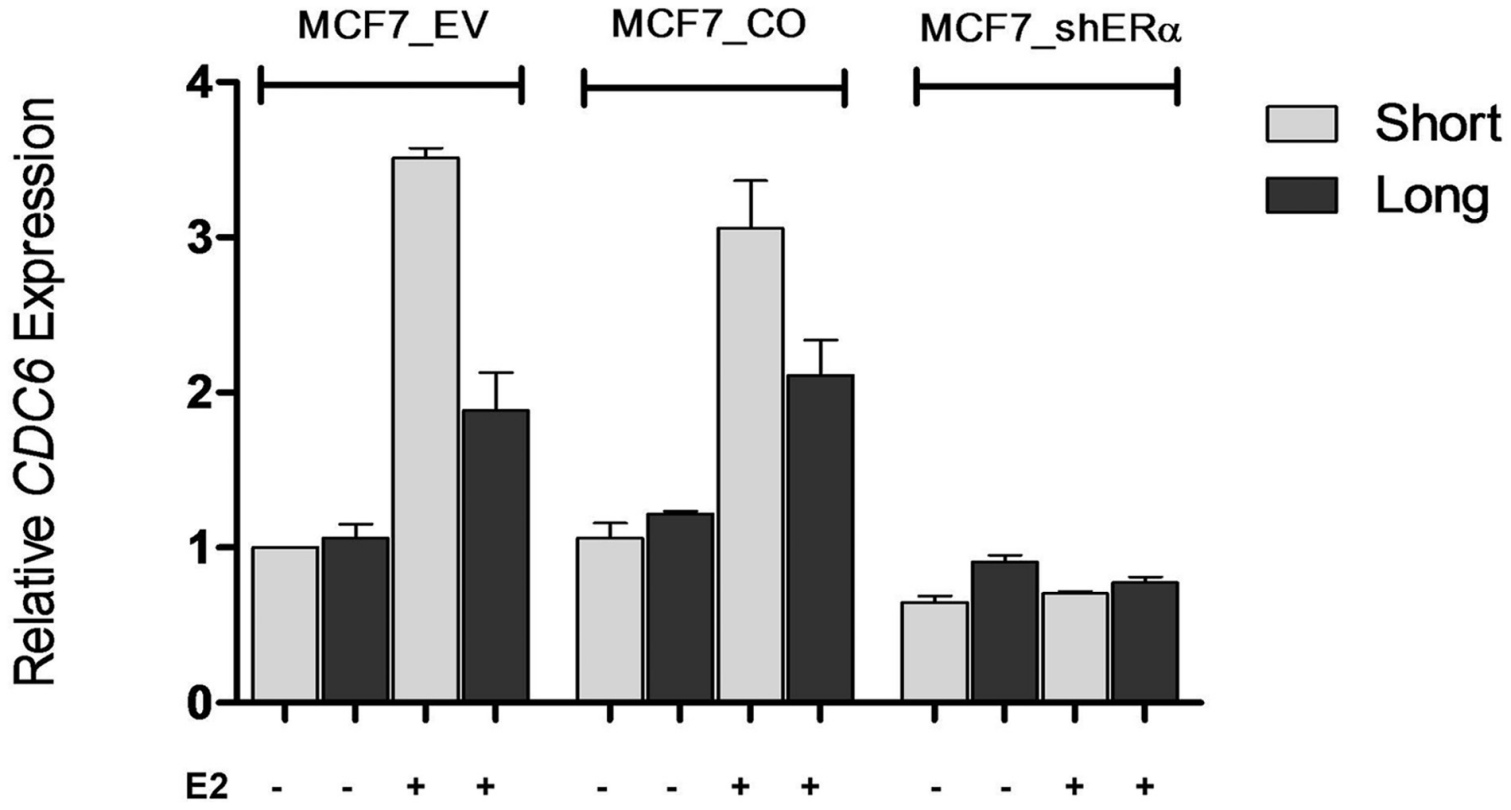
E2 does not induce *CDC6* 3′-UTR isoform increase in ER-silenced MCF7 cells. Cells were treated with E2 as described earlier. Relative quantification of *CDC6* 3′-UTR short and long isoforms was determined in MCF7-EV (empty vector transfected), MCF7_CO (control shRNA transfected) and in MCF7_shERα cells before and after 12 h of E2 treatment. The fold change for the isoforms was normalized against the reference gene, *SDHA. *Quantification was done using the reaction efficiency correction and ΔΔCq method (29). The baseline for the short and long isoforms in untreated samples was set to 1.

To understand whether increased abundance of the short 3′-UTR isoform compared with the long isoform was indeed due to transcriptional upregulation by E2 and ER, we treated cells with an ER-specific antagonist ICI 182,780 (ICI) (24) alone or together with E2. Based on our earlier E2 treatment time-points, cells were treated with ICI and E2 for 12 h. Although RT-qPCR results clearly showed increased short 3′-UTR isoform compared with the longer isoform in response to E2 alone, short and long *CDC6* expression decreased in ICI and E2-treated cells, showing the ER-specific response in MC7 and T47D cells (Figure 6A and B). ER-specific response was further investigated using MCF7 cells that were stably transfected with an shRNA construct to silence ER (Supplementary Figure S7). E2 treatment in ER-silenced MCF7 cells did not cause an increase in the *CDC6* short isoform whereas MCF7_EV (empty vector transfected) and MCF7_CO (control shRNA transfected) cells had increased *CDC6* short isoform expression in response to E2 (Figure 7). These results further strengthened the findings on E2 and ER dependency of *CDC6* APA.

Next, we explored whether *CDC6* was a primary target of ER. When cells were pre-treated with cycloheximide (CHX) (to block translation) and then with E2 for 12 h, the transcriptional upregulation of *CDC6* was prevented (Figure 6A and B), suggesting *CDC6* to be transcriptionally upregulated possibly by other transcription factors.

Of note, both short and long isoform transcription was demolished after ICI, and CHX treatments in ER+ cells, further confirming the role of E2-induced, ER-dependent transcription and APA regulation of *CDC6*. Effectiveness of the ICI and CHX treatments was confirmed with the diminished expression of *PCNA *(proliferating cell nuclear antigen) (Supplementary Figure S8). *PCNA* is a known secondary response gene that is upregulated in the presence of E2 (33).

E2-mediated response in ER-positive mammary cell lines is associated with the expression of a vast number of genes, some of which are involved in proliferation. It is therefore difficult to assess whether E2-mediated 3′-UTR shortening of *CDC6* is related to cell proliferation. To circumvent this issue, we used ERα expressing MDA-MB-231 cells (MDA-66) (19) (Supplementary Figure S7). In contrast to ER-positive MCF7 or T47D cells, re-expression of ERα endogenously or exogenously suppresses the growth of MDA-MB-231 cells in a time-dependent manner (34–37). Consistent with these studies, short-term E2 treatment (up to 18 h) did not cause an increase in *CDC6* 3′-UTR short isoform expression compared with long isoform (Supplementary Figure S9A) or BrdU incorporation (Supplementary Figure S9B), whereas E2-responsive *TFF1* transcript levels increased (Supplementary Figure S9C). These findings suggest that E2-mediated 3′-UTR shortening of *CDC6* is coupled to cellular proliferation.

The mechanism by which proliferation induces APA is unknown. Recent studies showed that E2F transcription factors are involved in the enhanced APA in proliferation (28). E2 treatment of MCF7 cells also increased the expression of *E2F* (*E2F1* and *E2F2*) transcription factors that are among regulators of *CDC6* (38,39) and 3′-end-processing proteins (28) such as *CSTF2* (cleavage stimulation factor, 3′ pre-RNA, subunit 2), *CSTF3* (cleavage stimulation factor, 3′ pre-RNA, subunit 3) and *CPSF2* (cleavage and polyadenylation-specific factor 2) (Supplementary Figure S10). Thus, E2-mediated increases in E2F along with 3′-end-processing enzyme levels may be responsible for APA in proliferation.

## DISCUSSION

There is growing evidence on the functional consequences of over-active DNA replication machinery contributing to transformation in cells. Recently, Di Paola *et al.* showed over-expression and increased chromatin-association of pre-replication complex proteins, one of which was CDC6, in transformed cells (40). CDC6 is a proto-oncogenic protein that contributes to oncogenesis through its role in DNA replication initiation and its repressive effect on the *INK4/ARF* locus (41,42). Interestingly, mechanisms to explain increased activation of CDC6 in cancer cells are not well known. Here, by using an *in silico* and *in vitro* approach, we show that *CDC6* is upregulated and its 3′-UTR is shortened in response to E2 in breast cancer cells. APA-dependent 3′-UTR shortening is gaining a well deserved attention due to its potential role in proto-oncogene activation cases where no other causative factor is known. To identify such 3′-UTR shortening cases, we took an *in silico* approach that allowed us to re-analyse existing gene expression arrays at the probe level based on the positions of the reported poly(A) sites. Although limited to array design decisions, we were able to group 3′-UTR matching probes into proximal and distal sets based on the poly(A) site positions. Using such probe sets, we found *CDC6* proximal and distal probe ratios to differentially increase in response to E2 in three independent datasets, suggesting APA regulation of *CDC6*. This approach allowed us to distinguish E2-induced differential expression of 3′-UTR isoforms. This observation emphasizes the importance of RNA sequencing and/or development of expression analysis systems that can facilitate detection of APA regulated and alternatively spliced isoforms which may be undetected in conventional gene expression array systems.

However, because such a probe-based approach may introduce certain quantitative biases, E2-induced 3′-UTR shortening of *CDC6* was further confirmed via *in vitro* assays. In accordance with the *in silico* analysis, E2 did induce upregulation of both isoforms, more so of the shorter isoform, in ER positive breast cancer cells. Moreover, upregulation of the short isoform correlated with increased CDC6 protein levels and BrdU incorporation which may reflect increased S phase entry. We further detected higher translation of the shorter isoform compared with the longer one in a luciferase reporter assay system. The reason of such 3′-UTR shortening of *CDC6* could be due to the need to rapidly upregulate protein levels upon a proliferation signal, in our case E2, by escaping from miRNA-dependent negative regulation. Interestingly, the 4 miRNAs predicted by different bioinformatics tools, were implicated in G1/S entry network and cell proliferation in the literature. Although expressed as part of different clusters, miR-25, miR-92a and miR-92b have the same seed sequence (AUUGCA) that is *in silico* predicted to bind to *CDC6*. miR-25 and miR-92b were shown to target p57, a Cip/Kip family member of Cdk inhibitors and thus may have control over G1/S transition (43,44). On the other hand, miR-541 was shown to play roles in differentiation, development and androgen-induced cell proliferation (45,46). The possible connection between these miRs, 3′-UTR shortening of *CDC6* and cell cycle regulation remains to be further investigated. Other mechanisms (e.g. adenylate–uridylate-rich *cis*-elements and/or RNA binding proteins) may also negatively affect the translation rate and/or subcellular localization of the long isoform. Although the upregulation and 3′-UTR shortening of *CDC6* were E2 and ER dependent, we also observed that the expression level of the longer isoform was decreased after the ICI and CHX treatments, independent of E2 (Figure 6A and B). The decreased levels of *CDC6* long isoform after inhibition of ER and protein synthesis may indicate the involvement of ER and possibly other transcription factors such as E2F (38,39) that may induce low level basal expression of the *CDC6* transcript. Interestingly, we also observed increased levels of *E2F1* and *E2F2* in response to E2. E2F transcription factors are not only involved in the regulation of G1/S entry proteins but also known to regulate 3′-UTR processing enzymes (28) which, in our study, showed upregulation in response to E2. Recently, 3′-UTR processing genes were found to be upregulated in proliferating cells and E2F was shown to mediate enhanced APA (28). Together, these findings may pioneer further studies to understand how APA is induced due to proliferative signals such as E2. To our knowledge, this study is the first to show hormone-regulated 3′-UTR shortening of a potential oncogene in cancer cells. As thoroughly reviewed, E2 action and miRNA regulation is already an attractive research area (47). Investigation of such E2-regulated APA events to avoid 3′-UTR-dependent negative regulations may further contribute toward elucidating a more comprehensive understanding of diverse responses to E2 and of the mechanisms of rapid proliferation in hormonally responsive cancer cells. Given that there is mounting evidence on the significance of APA as an overlooked mechanism of gene expression control in normal and in cancer cells, further studies are underway to determine proliferation-induced APA.

It also seems crucial to consider and explore APA in specific contexts where it may be controlled by different machinery and lead to different outcomes. For example, in E2-treated cells, APA of *CDC6* seemed to be due to the proliferative effect of E2. Proliferation signals (growth factors, hormones, etc.) or tissue origin may require and elucidate different APA mechanisms and responses in normal and in heterogeneous patient tumor samples. On the other hand, cancer cells may also favor APA to alter 3′-UTR lengths independent of the proliferative signals. Therefore, combinatorial use of different experimental and model systems is likely to give a more realistic picture of the extent of APA and 3′-UTR shortening events. A genome-wide polyadenylation mapping study based on high-throughput sequencing revealed that most yeast and human transcripts have as of yet uncharacterized and/or non-canonical polyadenylation sites, pointing out to the extent of complexity we are yet to face (48,49). Given the significance of these new findings, we are likely to better understand not only how gene expression is regulated by APA but also how different choices of APA may further be involved in different physiological and disease state.

## SUPPLEMENTARY DATA

Supplementary Data are available at NAR Online: Supplementary Tables 1–4 and Supplementary Figures 1–10.

## FUNDING

ODTU (Orta Dogu Teknik Universitesi-METU [Middle East Technical University]); intramural interdisciplinary research funds [DAP2010/2011]. Funding for open access charge: European Union in the 7th framework program through SysPatho [260429, in part].

*Conflict of interest statement*. None declared.

## Supplementary Material

Supplementary Data
