# Supplementary material for: Estrogen-induced upregulation and 3′-UTR shortening of CDC6
Source: Nucleic Acids Res. 2012 Sep 12;40(21):10679–88. doi: 10.1093/nar/gks855 (PMC3510512; doi:10.1093/nar/gks855)
Supplement: Supplementary Data [file supp_40_21_10679__index.html]

Estrogen-induced upregulation and 3′-UTR shortening of CDC6 — Estrogen-induced upregulation and 3′-UTR shortening of CDC6 — Supplementary Data 

# Estrogen-induced upregulation and 3′-UTR shortening of *CDC6*

## Supplementary Data

files

**Files in this Data Supplement:**

- Supplementary Data - pdf file
