# Supplementary material for: Estrogen-induced upregulation and 3′-UTR shortening of CDC6
Source: Nucleic Acids Res. 2012 Sep 12;40(21):10679–88. doi: 10.1093/nar/gks855 (PMC3510512; doi:10.1093/nar/gks855)
Supplement: Supplementary Data [file supp_gks855_nar-01248-n-2012-File013.pdf]

Supplementary Table 1. MIQE Guidelines Checklist

ITEM TO CHECK	IMPORTANCE	CHECKLIST	COMMENTS/ WHERE?
**EXPERIMENTAL DESIGN**			
Definition of experimental and control groups	**E**	**YES**	Materials and Methods
Number within each group	**E**	**YES**	Materials and Methods
Assay carried out by core lab or investigator's lab?	D	**YES**	Investigator's Lab
Acknowledgement of authors' contributions	D	**NO**	Not required by journal
**SAMPLE**			
Description	**E**	**N/A**	
Volume/mass of sample processed	D	**N/A**	
Microdissection or macrodissection	**E**	**N/A**	
Processing procedure	**E**	**N/A**	
If frozen - how and how quickly?	**E**	**N/A**	
If fixed - with what, how quickly?	**E**	**N/A**	
Sample storage conditions and duration (especially for FFPE samples)	**E**	**N/A**	
**NUCLEIC ACID EXTRACTION**			
Procedure and/or instrumentation	**E**	**YES**	Materials and Methods
Name of kit and details of any modifications	**E**	**YES**	Materials and Methods
Source of additional reagents used	D	**YES**	Materials and Methods
Details of DNase or RNAse treatment	**E**	**YES**	Sup. Table 2
Contamination assessment (DNA or RNA)	**E**	**YES**	Sup. Fig. 1
Nucleic acid quantification	**E**	**YES**	Materials and Methods Sup. Fig. 1
Instrument and method	**E**	**YES**	Materials and Methods Sup. Fig. 1
Purity (A260/A280)	D	**YES**	Sup. Fig. 1
Yield	D	**YES**	Sup. Fig. 1
RNA integrity method/instrument	**E**	**YES**	Agarose gel Sup. Fig. 1
RIN/RQI or Cq of 3' and 5' transcripts	**E**	**NO**	
Electrophoresis traces	D	**N/A**	
Inhibition testing (Cq dilutions, spike or other)	**E**	**YES**	Cq dilutions Sup. Fig. 3
**REVERSE TRANSCRIPTION**			
Complete reaction conditions	**E**	**YES**	Sup. Table 3

Amount of RNA and reaction volume	**E**	**YES**	Materials and Methods Sup. Table 3
Priming oligonucleotide and concentration	**E**	**YES**	Materials and Methods Sup. Table 3
Reverse transcriptase and concentration	**E**	**YES**	Sup. Table 3
Temperature and time	**E**	**YES**	Sup. Table 3
Manufacturer of reagents and catalogue numbers	D	**YES**	Sup. Table 3
Cqs with and without RT	D	**NO**	
Storage conditions of cDNA	D	**YES**	Sup. Table 3
**qPCR TARGET INFORMATION**			
If multiplex, efficiency and LOD of each assay.	**E**	**N/A**	
Sequence accession number	**E**	**YES**	CDC6:NM_001254 SDHA:NM_004168
Location of amplicon	D	**YES**	Sup. Figure 2
Amplicon length	**E**	**YES**	Materials and Methods Sup. Figure 2
In silico specificity screen (BLAST, etc)	**E**	**YES**	BLAST and *In silico* PCR Sup. Figure 2
Pseudogenes, retropseudogenes or other homologs?	D	**YES**	None detected by BLASTn
Sequence alignment	D	**YES**	Sup. Figure 2
Secondary structure analysis of amplicon	D	**NO**	
Location of each primer by exon or intron (if applicable)	**E**	**YES**	Sup. Figure 2
What splice variants are targeted?	**E**	**N/A**	
**qPCR OLIGONUCLEOTIDES**			
Primer sequences	**E**	**YES**	Materials and Methods
RTPrimerDB Identification Number	D	**N/A**	
Probe sequences	D	**N/A**	
Location and identity of any modifications	**E**	**N/A**	
Manufacturer of oligonucleotides	D	**YES**	Materials and Methods
Purification method	D	**YES**	HPLC

**qPCR PROTOCOL**			
Complete reaction conditions	**E**	**YES**	Materials and Methods Sup. Table 4
Reaction volume and amount of cDNA/DNA	**E**	**YES**	Materials and Methods Sup. Table 3
Primer, (probe), Mg++ and dNTP concentrations	**E**	**YES**	Materials and Methods
Polymerase identity and concentration	**E**	**YES**	Materials and Methods
Buffer/kit identity and manufacturer	**E**	**YES**	Materials and Methods
Exact chemical constitution of the buffer	D	**NO**	Manufactures proprietary
Additives (SYBR Green I, DMSO, etc.)	**E**	**N/A**	
Manufacturer of plates/tubes and catalog number	D	**YES**	Sup. Table 3
Complete thermocycling parameters	**E**	**YES**	Materials and Methods
Reaction setup (manual/robotic)	D	**YES**	Manual setup
Manufacturer of qPCR instrument	**E**	**YES**	Materials and Methods
**qPCR VALIDATION**			
Evidence of optimization (from gradients)	D		
Specificity (gel, sequence, melt, or digest)	**E**	**YES**	Melt analysis Sup. Figure 3
For SYBR Green I, Cq of the NTC	**E**	**YES**	Sup. Figure 3
Standard curves with slope and y-intercept	**E**	**YES**	Sup. Figure 3
PCR efficiency calculated from slope	**E**	**YES**	Sup. Figure 3
Confidence interval for PCR efficiency or standard error	D	**N/A**	
r2 of standard curve	**E**	**YES**	Sup. Figure 3
Linear dynamic range	**E**	**YES**	Sup. Figure 3
Cq variation at lower limit	**E**	**YES**	Sup. Figure 3
Confidence intervals throughout range	D	**N/A**	
Evidence for limit of detection	**E**	**NO**	
If multiplex, efficiency and LOD of each assay.	**E**	**N/A**	
**DATA ANALYSIS**			
qPCR analysis program (source, version)	**E**	**YES**	Materials and Methods
Cq method determination	**E**	**YES**	Materials and Methods
Outlier identification and disposition	**E**	**N/A**	
Results of NTCs	**E**	**YES**	Sup. Figure 3
Justification of number and choice of reference genes	**E**	**YES**	Materials and Methods
Description of normalization method	**E**	**YES**	Standard curve quantification
Number and concordance of biological replicates	D	**YES**	Materials and

			Methods
Number and stage (RT or qPCR) of technical replicates	**E**	**YES**	Materials and Methods
Repeatability (intra-assay variation)	E	**YES**	Materials and Methods
Reproducibility (inter-assay variation, %CV)	D	**NO**	
Power analysis	D	**NO**	
Statistical methods for result significance	**E**	**YES**	Biological replicates
Software (source, version)	E	**YES**	Materials and Methods
Cq or raw data submission using RDML	**D**	**N/A**	

E: Essential information, D: Desirable information, N/A: Not applicable

Supplementary Table 2. DNase Treatment

Components	Amount
Total RNA	10 µg
10X Incubation Buffer<br>(400 mM Tris-HCl, 100 mM NaCl, 60 mM $MgCl_2$, 10 mM $CaCl_2$, pH 7.9)	10 µL
DNase I recombinant, RNase-free (10 units/µl)<br>Roche Applied Sciences Cat. No. 04 716 728 001	1 µL
RiboLock™ RNase Inhibitor (20u/µL)<br>Fermentas #EO0381<br>*One unit of RiboLock™ RNase Inhibitor inhibits the activity of 5 ng RNase A by 50%	1 µL
RNase-free Water	To 100 µL
DNase treatment was done at 37º C for 60 min. Reaction was terminated by phenol/chloroform extraction.	

Supplementary Table 3. Reaction Conditions of Reverse Transcription

Components	Amount
Total RNA	2 µg
Oligo(dT)$_{18}$ primer (100 µM 0.5 µg/µl (15 A260 u/ml))	1 µL
5X Reaction Buffer<br>(250 mM Tris-HCl (pH 8.3), 250 mM KCl, 20 mM $MgCl_2$, 50 mM DTT)	4 µL
10 mM dNTP mix	2 µL
RiboLock™ RNase Inhibitor (20u/µL)<br>*One unit of RiboLock™ RNase Inhibitor inhibits the activity of 5 ng RNase A by 50%	1 µL
RevertAid™ M-MuLV Reverse Transcriptase (200u/µL)<br>*One unit of RevertAid™ M-MuLV RT incorporates 1 nmol of dTMP into a polynucleotide fraction (adsorbed on DE-81) in 10 min at 37°C	1 µL
Nuclease-free water	to 20 µL
cDNA was synthesized at 42º C for 60 minutes. Reaction was terminated by keeping at 70º C for 5 minutes. cDNA samples were stored at -20º C.	

Supplementary Table 4. Reaction Conditions for RT-qPCR

Components	Amount
Fast Start SYBR Green Master	10 μL
Forward Primer (5 μM)	1.2 μL
Reverse Primer (5 μM)	1.2 μL
cDNA (1:49 diluted from stock cDNA)	4 μL
PCR Grade water	3.6 μL
Total volume	20 μL
PCR tubes (0.2 mL, thin wall, flat cap, Greiner catalog number Z617687).	

A.

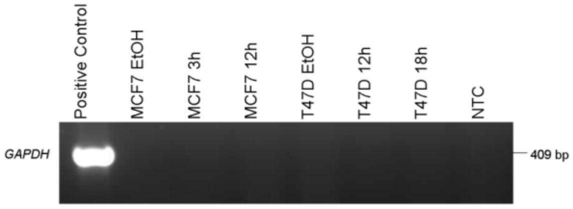

B.

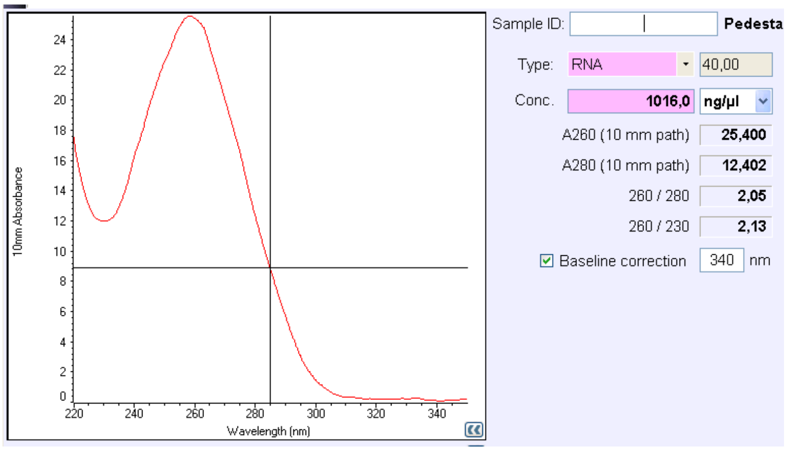

C.

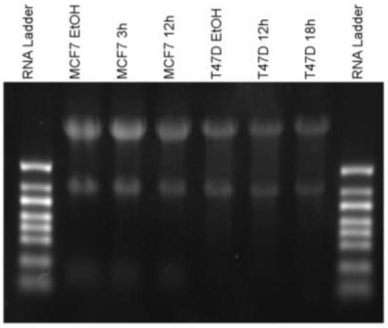

Supplementary Figure 1. DNA contamination, quantification and integrity assessment

A. Lack of DNA contamination in RNA samples was assessed and shown by PCR using *GAPDH* specific primers. GAPDH_F: 5'-GGGAGCCAAAAGGGTCATCA-3' and GAPDH_R: 5'-TTTCTAGACGGCAGGTCA GGT-3' (product size: 409 bp). Following conditions were used for the PCR reactions: incubation at 94^{0}C for 10 minutes, 40 cycles of 94^{0}C for 30 seconds, 56^{0}C for 30 seconds, and 72^{0}C for 30 seconds, and final extension at 72^{0}C for 5 minutes. MCF7 cDNA was used as a positive control. **B.** RNA concentrations (A260) were determined using NanoDrop ND1000 (Thermo Scientific). Purity was determined by A260/A280 and A260/A230 ratios (all RNA samples were around 2.0 for A260/A280, and A260/A230 ratios) **C.** RNA integrity was assessed by running the RNA samples on a 0.7 % Formaldehyde RNA Gel (10X MOPS, 37% Formaldehyde, DEPC-water)

A.

```
>ref|NM_001254.3| UEGM Homo sapiens cell division cycle 6 homolog (S. cerevisiae) (CDC6),
mRNA
Length=3053

 GENE ID: 990 CDC6 | cell division cycle 6 homolog (S. cerevisiae)
[Homo sapiens] (Over 10 PubMed links)


 Score =  342 bits (185),  Expect = 9e-92
 Identities = 185/185 (100%), Gaps = 0/185 (0%)
 Strand=Plus/Plus

Query  1     TTCAGCTGGCATTTAGAGAGCTACAGTCTTCATTTTAGTGCTTTACACATTCGGGCCTGA  60
             ||||||||||||||||||||||||||||||||||||||||||||||||||||||||||||
Sbjct  1923  TTCAGCTGGCATTTAGAGAGCTACAGTCTTCATTTTAGTGCTTTACACATTCGGGCCTGA  1982

Query  61    AAACAAATATGACCTTTTTTACTTGAAGCCAATGAATTTTAATCTATAGATTCTTTAATA  120
             ||||||||||||||||||||||||||||||||||||||||||||||||||||||||||||
Sbjct  1983  AAACAAATATGACCTTTTTTACTTGAAGCCAATGAATTTTAATCTATAGATTCTTTAATA  2042

Query  121   TTAGCACAGAATAATATCTTTGGGTCTTACTATTTTTACCCATAAAAGTGACCAGGTAGA  180
             ||||||||||||||||||||||||||||||||||||||||||||||||||||||||||||
Sbjct  2043  TTAGCACAGAATAATATCTTTGGGTCTTACTATTTTTACCCATAAAAGTGACCAGGTAGA  2102

Query  181   CCCTT  185
             |||||
Sbjct  2103  CCCTT  2107
```

UCSC In-Silico PCR

```
>chr17:38458283+38458467 185bp TTCAGCTGGCATTTAGAGAGC AAGGGTCTACCTGGTCACTTTT
TTCAGCTGGCATTTAGAGAGCtacagtcttcattttagtgctttacacat
tcgggcctgaaaacaaatatgaccttttttacttgaagccaatgaatttt
aatctatagattctttaatattagcacagaataatatctttgggtcttac
tatttttacccatAAAAGTGACCAGGTAGACCCTT
```

Primer Melting Temperatures

Forward: 59.7 C ttcagctggcatttagagagc
Reverse: 58.2 C aagggtctacctggtcactttt
The temperature calculations are done assuming 50 mM salt and 50 nM annealing oligo concentration. The code to calculate the melting temp comes from Primer3.

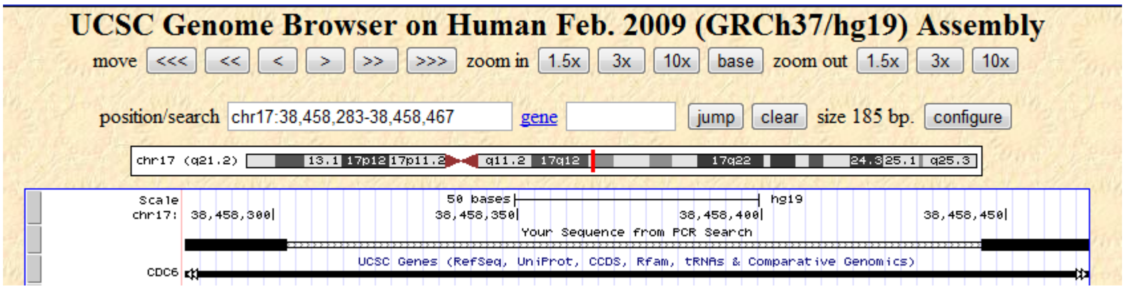

B.

```
>ref|NM_001254.3| UEGM Homo sapiens cell division cycle 6 homolog (S. cerevisiae) (CDC6),
mRNA
Length=3053

 GENE ID: 990 CDC6 | cell division cycle 6 homolog (S. cerevisiae)
[Homo sapiens] (Over 10 PubMed links)

 Score =  645 bits (349),  Expect = 0.0
 Identities = 349/349 (100%), Gaps = 0/349 (0%)
 Strand=Plus/Plus

Query  1     TTCAGCTGGCATTTAGAGAGCTACAGTCTTCATTTTAGTGCTTTACACATTCGGGCCTGA  60
             ||||||||||||||||||||||||||||||||||||||||||||||||||||||||||||
Sbjct  1923  TTCAGCTGGCATTTAGAGAGCTACAGTCTTCATTTTAGTGCTTTACACATTCGGGCCTGA  1982

Query  61    AAACAAATATGACCTTTTTTACTTGAAGCCAATGAATTTTAATCTATAGATTCTTTAATA  120
             ||||||||||||||||||||||||||||||||||||||||||||||||||||||||||||
Sbjct  1983  AAACAAATATGACCTTTTTTACTTGAAGCCAATGAATTTTAATCTATAGATTCTTTAATA  2042

Query  121   TTAGCACAGAATAATATCTTTGGGTCTTACTATTTTTACCCATAAAAGTGACCAGGTAGA  180
             ||||||||||||||||||||||||||||||||||||||||||||||||||||||||||||
Sbjct  2043  TTAGCACAGAATAATATCTTTGGGTCTTACTATTTTTACCCATAAAAGTGACCAGGTAGA  2102

Query  181   CCCTTTTTAATTACATTCACTACTTCTACCACTTGTGTATCTCTAGCCAATGTGCTTGCA  240
             ||||||||||||||||||||||||||||||||||||||||||||||||||||||||||||
Sbjct  2103  CCCTTTTTAATTACATTCACTACTTCTACCACTTGTGTATCTCTAGCCAATGTGCTTGCA  2162

Query  241   AGTGTACAGATCTGTGTAGAGGAATGTGTGTATATTTACCTCTTCGTTTGCTCAAACATG  300
             ||||||||||||||||||||||||||||||||||||||||||||||||||||||||||||
Sbjct  2163  AGTGTACAGATCTGTGTAGAGGAATGTGTGTATATTTACCTCTTCGTTTGCTCAAACATG  2222

Query  301   AGTGGGTATTTTTTTGTTTGTTTTTTTTGTTGTTGTTGTTTTTGAGGCG  349
             |||||||||||||||||||||||||||||||||||||||||||||||||
Sbjct  2223  AGTGGGTATTTTTTTGTTTGTTTTTTTTGTTGTTGTTGTTTTTGAGGCG  2271
```

UCSC In-Silico PCR

```
>chr17:38458283+38458631 349bp TTCAGCTGGCATTTAGAGAGC CGCCTCAAAAACAACAACAA
TTCAGCTGGCATTTAGAGAGCtacagtcttcattttagtgctttacacat
tcgggcctgaaaacaaatatgaccttttttacttgaagccaatgaatttt
aatctatagattctttaatattagcacagaataatatctttgggtcttac
tatttttacccataaaagtgaccaggtagacccttttttaattacattcac
tacttctaccacttgtgtatctctagccaatgtgcttgcaagtgtacaga
tctgtgtagaggaatgtgtgtatatttacctcttcgtttgctcaaacatg
agtgggtatttttttgtttgttttttttgTTGTTGTTGTTTTTGAGGCG
```

Primer Melting Temperatures

Forward: 59.7 C ttcagctggcatttagagagc
Reverse: 59.7 C cgcctcaaaaacaacaacaa
The temperature calculations are done assuming 50 mM salt and 50 nM annealing oligo concentration. The code to calculate the melting temp comes from Primer3.

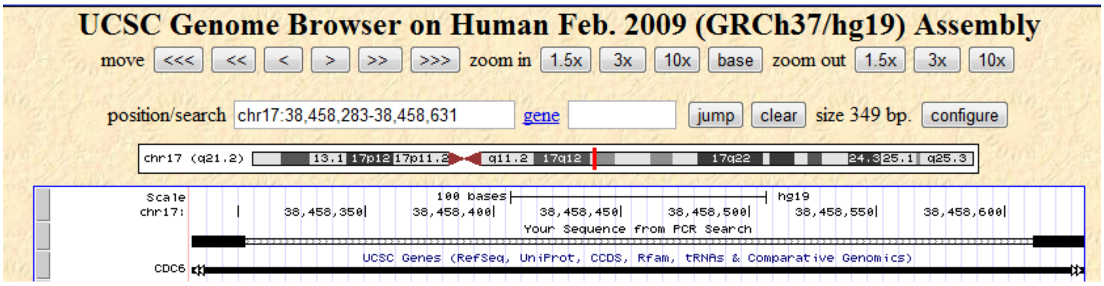

C.

```
>ref|NM_004168.2| UEGM Homo sapiens succinate dehydrogenase complex, subunit A, flavoprotein
(Fp) (SDHA), nuclear gene encoding mitochondrial protein,
mRNA
Length=2405

 GENE ID: 6389 SDHA | succinate dehydrogenase complex, subunit A, flavoprotein
(Fp) [Homo sapiens] (Over 10 PubMed links)


 Score =  159 bits (86),  Expect = 3e-37
 Identities = 86/86 (100%), Gaps = 0/86 (0%)
 Strand=Plus/Plus

Query  1    TGGGAACAAGAGGGCATCTGCTAAAGTTTCAGATTCCATTTCTGCTCAGTATCCAGTAGT  60
            ||||||||||||||||||||||||||||||||||||||||||||||||||||||||||||
Sbjct  229  TGGGAACAAGAGGGCATCTGCTAAAGTTTCAGATTCCATTTCTGCTCAGTATCCAGTAGT  288

Query  61   GGATCATGAATTTGATGCAGTGGTGG  86
            ||||||||||||||||||||||||||
Sbjct  289  GGATCATGAATTTGATGCAGTGGTGG  314
```

Supplementary Figure 2. ***In silico*** **specificity screens.**

In silico specificity of PCRs was assessed by BLAST (NCBI), amplicon lengths were confirmed using USCS *in silico* PCR tool, and chromosomal locations of amplicons are shown with Genome Browser of USCS for **A.** *CDC6* Short primers, **B.** *CDC6* Long primers, and **C.** *SDHA* primers (*SDHA* primers were designed from exon-exon boundaries)

A.

Melt Analysis for CDC6_Short primers

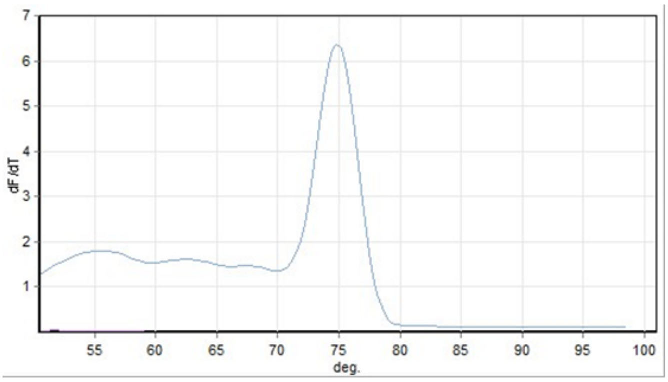

No.	Colour	Name	Genotype	Peak 1	Peak 2	Peak 3	Peak 4	Peak 5	Peak 6	Peak 7	Peak 8	Peak 9	Peak 10
1		CDC6 Short		55,5	62,5	67,5	74,8	87,0	97,0				
2		CDC6 Short NTC		51,7	57,0	61,7	67,2	72,7	76,0	81,8	87,0	92,5	97,0

Raw Data For Cycling A.Green

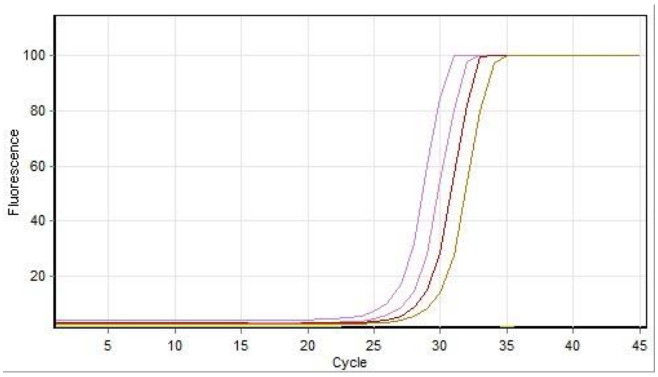

Quantitation data for Cycling A.Green

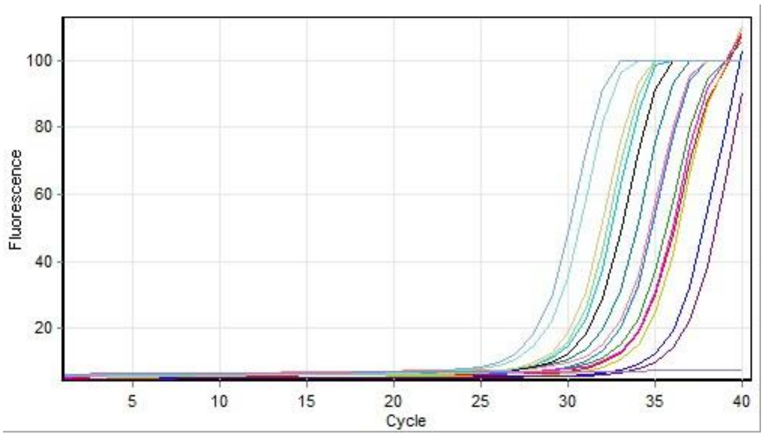

Standard Curve

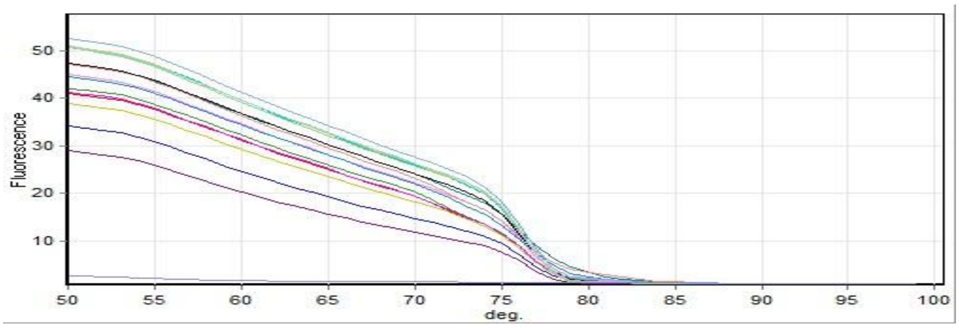

No.	Colour	Name	Type	Ct	Given Conc (copies/ul)	Calc Conc (copies/ul)	% Var
18		Std 1_CDC6 Short	Standard	22,84	100.000	100.501	0,5%
19		Std 2_CDC6 Short	Standard	23,85	50.000	48.956	2,1%
20		Std 3_CDC6 Short	Standard	24,75	25.000	25.689	2,8%
21		Std 4_CDC6 Short	Standard	25,77	12.500	12.362	1,1%
34		NTC_CDC6 Short	NTC				

Quantitation Information

Threshold	0,1232
Left Threshold	1,000
Standard Curve Imported	No
Standard Curve (1)	conc= 10^(-0,311*CT + 12,108)
Standard Curve (2)	CT = -3,215*log(conc) + 38,925
Reaction efficiency (*)	1,04669 (* = 10^(-1/m) - 1)
M	-3,21489
B	38,92467
R Value	0,99972
R^2 Value	0,99945
Start normalising from cycle	1
Noise Slope Correction	No
No Template Control Threshold	0%
Reaction Efficiency Threshold	Disabled
Normalisation Method	Dynamic Tube Normalisation
Digital Filter	Light
Sample Page	Page 1
Imported Analysis Settings	

B. **Melt Analysis for CDC6_Long primers**

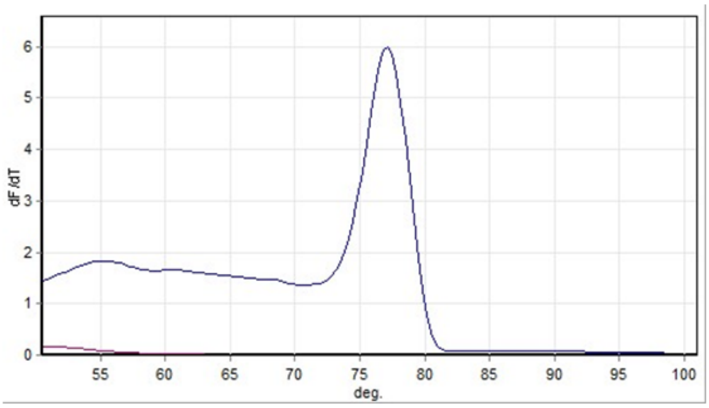

No.	Colour	Name	Genotype	Peak 1	Peak 2	Peak 3	Peak 4	Peak 5	Peak 6
1		CDC6 Long		55,3	60,5	77,0	83,0	90,3	95,5
2		CDC6 Long NTC		68,0	74,8	82,5	87,0	91,2	94,5

Raw Data For Cycling A.Green

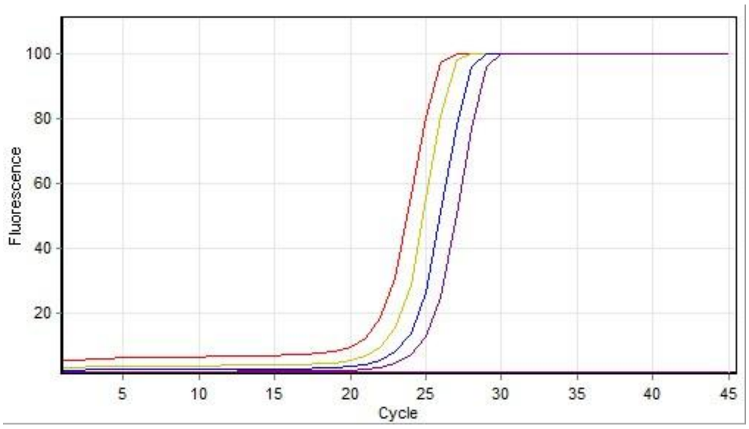

Quantitation data for Cycling A.Green

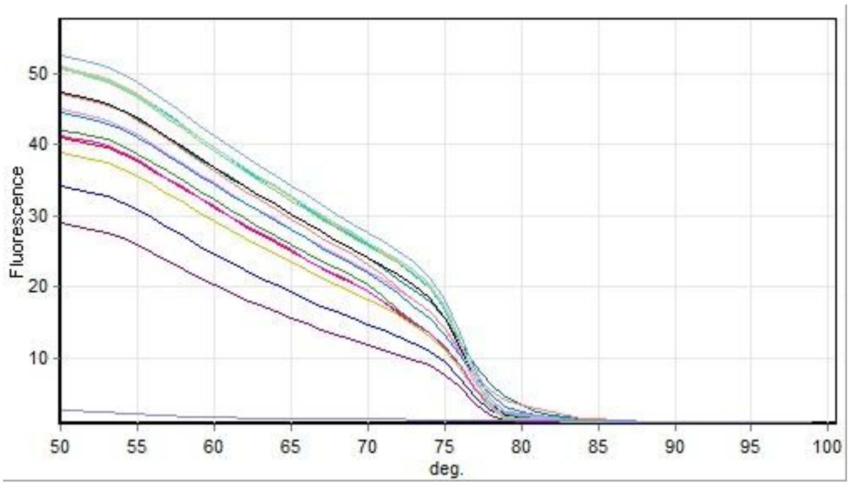

Standard Curve

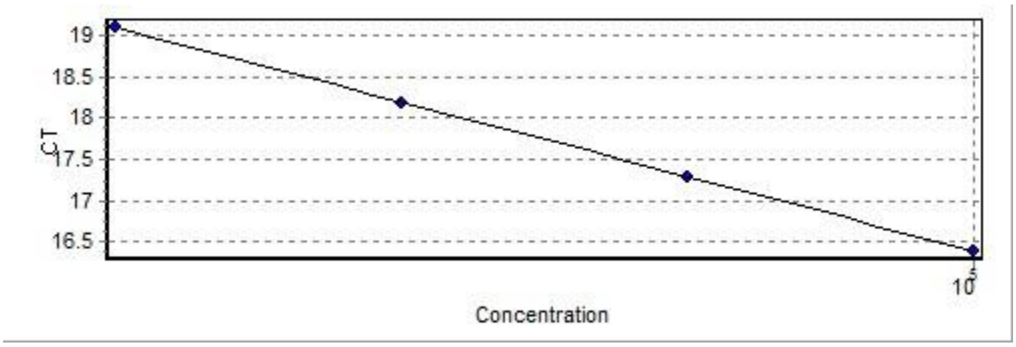

No.	Colour	Name	Type	Ct	Given Conc (copies/ul)	Calc Conc (copies/ul)	% Var
1		Std 1_CDC6 Long	Standard	16,39	100.000	99.959	0,0%
2		Std 2_CDC6 Long	Standard	17,29	50.000	49.894	0,2%
3		Std 3_CDC6 Long	Standard	18,19	25.000	25.137	0,5%
4		Std 4_CDC6 Long	Standard	19,10	12.500	12.463	0,3%
36		NTC	NTC				

Quantitation Information

Threshold	0,0554
Left Threshold	1,000
Standard Curve Imported	No
Standard Curve (1)	conc= 10^(-0,333*CT + 10,465)
Standard Curve (2)	CT = -2,999*log(conc) + 31,384
Reaction efficiency (*)	1,15494 (* = 10^(-1/m) - 1)
M	-2,99908
B	31,3844
R Value	0,99999
R^2 Value	0,99998
Start normalising from cycle	1
Noise Slope Correction	No
No Template Control Threshold	0%
Reaction Efficiency Threshold	Disabled
Normalisation Method	Dynamic Tube Normalisation
Digital Filter	Light
Sample Page	Page 1
Imported Analysis Settings	

C.

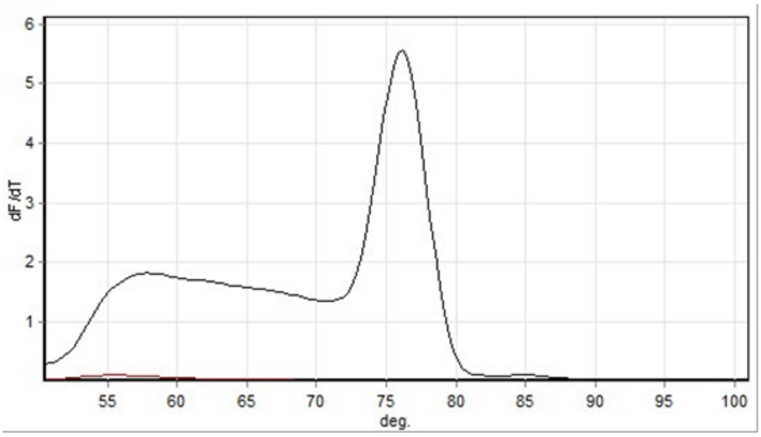

No.	Colour	Name	Genotype	Peak 1	Peak 2	Peak 3	Peak 4	Peak 5	Peak 6	Peak 7
1	■	SDHA		58,0	76,0	85,0				
2	■	SDHA NTC		55,3	66,7	75,3	81,5	85,5	90,3	96,5

Raw Data For Cycling A.Green

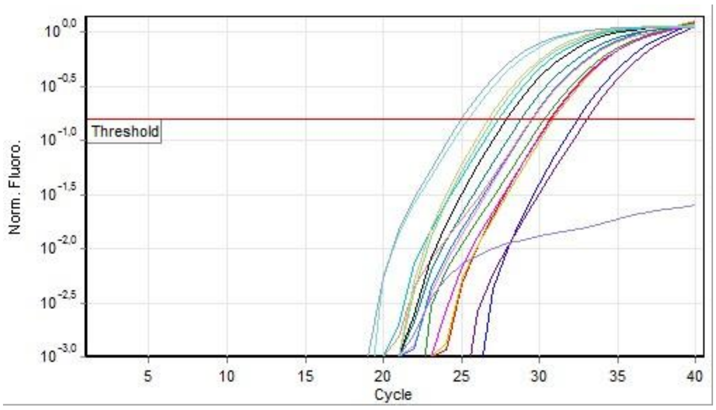

Quantitation data for Cycling A.Green

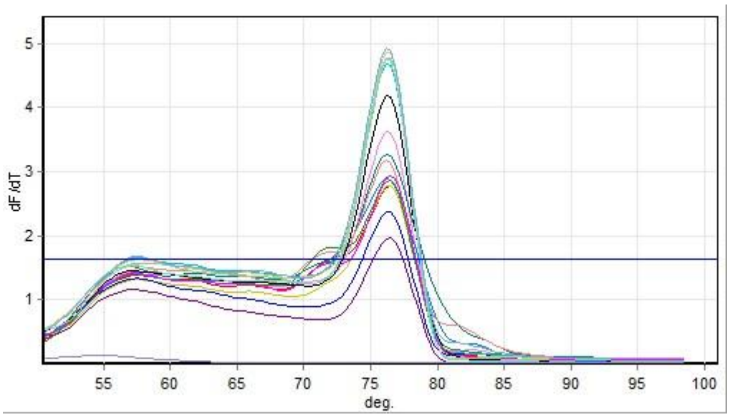

Standard Curve

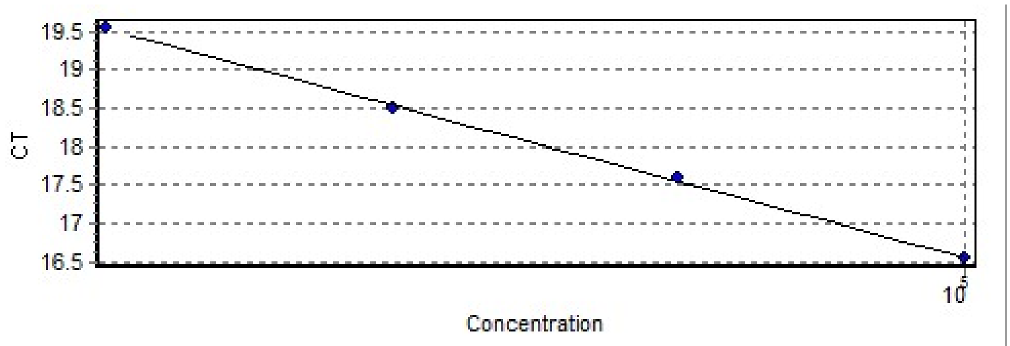

No.	Colour	Name	Type	Ct	Given Conc (copies/ul)	Calc Conc (copies/ul)	% Var
1		Std 1	Standard	16,54	100.000	100.703	0,7%
2		Std 2	Standard	17,58	50.000	48.899	2,2%
3		Std 3	Standard	18,50	25.000	25.594	2,4%
4		Std 4	Standard	19,54	12.500	12.397	0,8%
5		NTC	NTC				

Quantitation Information

Threshold	0,111
Left Threshold	1,000
Standard Curve Imported	No
Standard Curve (1)	conc= 10^(-0,303*CT + 10,019)
Standard Curve (2)	CT = -3,298*log(conc) + 33,039
Reaction efficiency (*)	1,01023 (* = 10^(-1/m) - 1)
M	-3,29766
B	33,03911
R Value	0,99976
R^2 Value	0,99952
Start normalising from cycle	1
Noise Slope Correction	No
No Template Control Threshold	0%
Reaction Efficiency Threshold	Disabled
Normalisation Method	Dynamic Tube Normalisation
Digital Filter	Light
Sample Page	Page 1
Imported Analysis Settings	

Supplementary Figure 3. RT-qPCR assay performance in accordance with MIQE guidelines.

Specificity of primers and the amplified PCR products was examined by melt analysis, raw data, quantitation data, standard curves, and quantitation information calculated by Rotor Gene Software for **A.** *CDC6* Short primers, **B.** *CDC6* Long primers, and **C.** *SDHA* primers. PCR efficiencies were calculated from slope, and r^2.

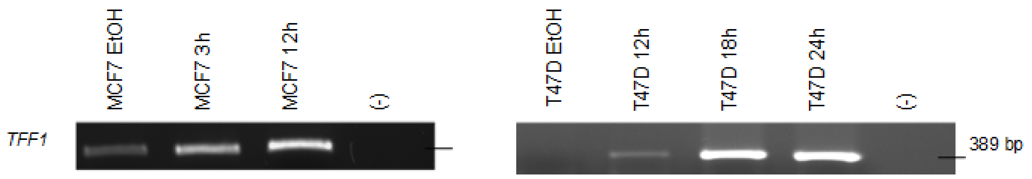

Supplementary Figure 4. *TFF1* is an E2 upregulated gene.

MCF7 and T47D cells were treated with ethanol or 10 nM E2 for indicated time points and RNA was isolated. Following DNase treatment, cDNA was synthesized for RT-PCR analysis. E2 treatment resulted with the upregulation of *TFF1* expression detected by RT-PCR

CDC6 1959 AGTGCTTTACACATTCGGGCCTGAAAACAAATATGACCTTTTTTACTTGAAGCCAATGAA

CDC6 2019 TTTTAATCTATAGATTCTTTAATATTAGCACAGAATAATATCTTTGGGTCTTACTATTTT

CDC6 2079 TACCCATAAAAGTGACCAGGTAGACCCTTTTTAATTACATTCACTACTTCTACCACTTGT

CDC6 2139 GTATCTCTAGCCAATGTGCTTGCAAGTGTACAGATCTGTGTAGAGGAATGTGTGTATATT

CDC6 2199 TACCTCTTCGTTTGCTCAAACATGAGTGGGTATTTT**T**TTTGTNN**AAAAAAAAAAAAAA**N

P-pA-Site

Supplementary Figure 5. Sequencing of the 3' RACE product (size: 355 bp) revealed the existence of the proximal polyA tail.

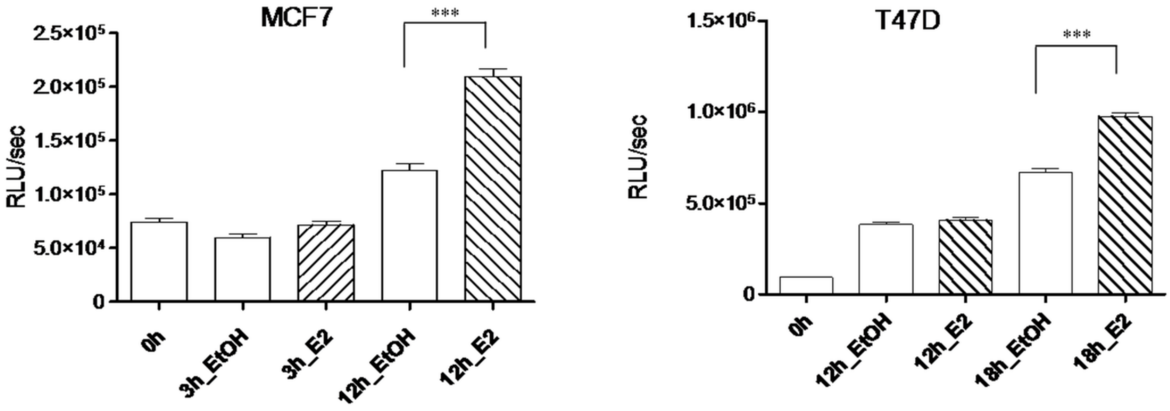

Supplementary Figure 6. E2 treatment induced increased BrdU incorporation. Asynchronous MCF7 and T47D cells were grown in phenol red-free media supplemented with 10% dextran-coated-charcoal stripped FBS for 48 hours. Then, cells were treated with BrdU (10 nM) and E2 (10 nM) for the indicated time points along with the ethanol treated controls (EtOH). Incorporated BrdU was detected using the Turner Biosystems Luminometer. Values are given as relative light units (RLU) per sec at respective time points. *** indicates statistical significance, $p<0.001$ (One way ANOVA followed by Tukey's multiple comparison test).

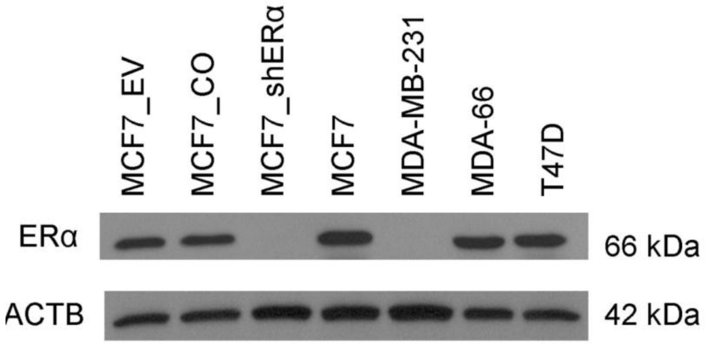

Supplementary Figure 7. ER protein levels detected in cell lines used in the study. MCF7_EV cells were stably transfected with empty vector (pSR), MCF7_CO cells were transfected with control shRNA (20), and MCF7_shERα cells were transfected with ERα shRNA (18). MDA-66 cells are MDA-MB-231 cells that were stably transfected with ER (18, 19). MCF7, MDA-MB-231 and T47D cells were used throughout the study as ER+ cells. 50 ug lysate were loaded on 8% PAGE. β-actin was used as a protein loading control.

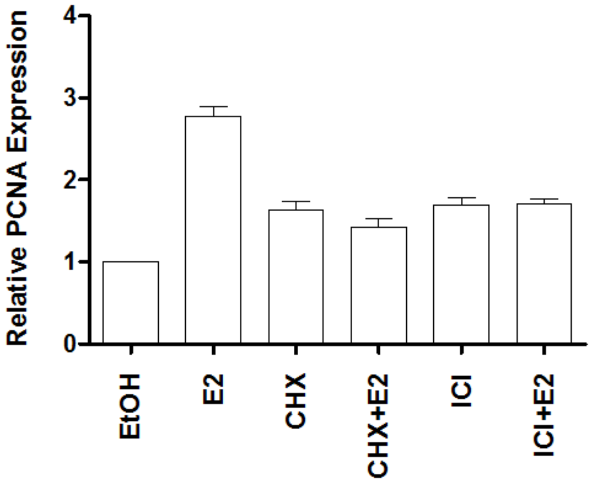

Supplementary Figure 8. *PCNA* expression detected by RT-qPCR after ICI and CHX treatments. MCF7 cells were grown in phenol red-free medium supplemented with 10% dextran-coated-charcoal stripped FBS, pre-treated with 1 µM ICI or 10 µg/mL CHX (Cycloheximide) for 1 hour, then with 10 nM E2 for 12 hours. The baseline for the control treated samples was set to 1. *PCNA* (NM_002592) was amplified using the following primer set; PCNA_F: 5'-TGCAGATGTACCCCTTGTTG-3', PCNA_R: 5'-GCTGGCATCTTAGAAGCAGTT-3'. Following conditions were used: incubation at 94°C for 10 minutes, 40 cycles of 94°C for 15 seconds, 56°C for 30 seconds, and 72°C for 30 seconds.

A.

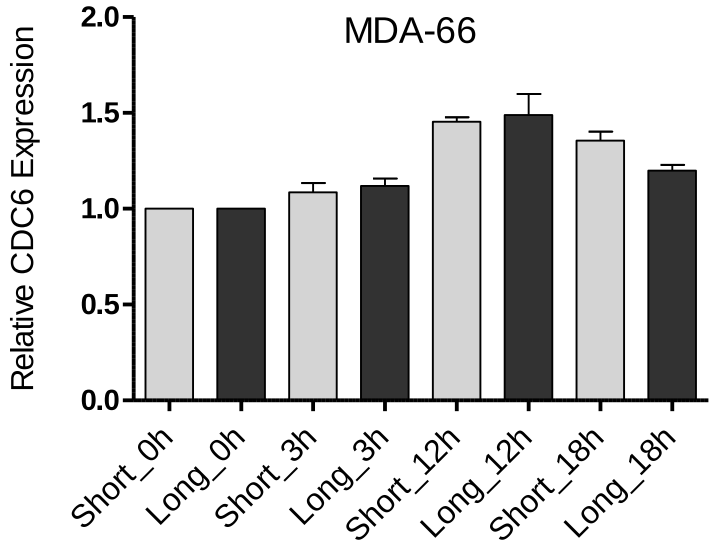
MDA-66
Relative CDC6 Expression
2.0
1.5
1.0
0.5
0.0
Short_0h
Long_0h
Short_3h
Long_3h
Short_12h
Long_12h
Short_18h
Long_18h

B.

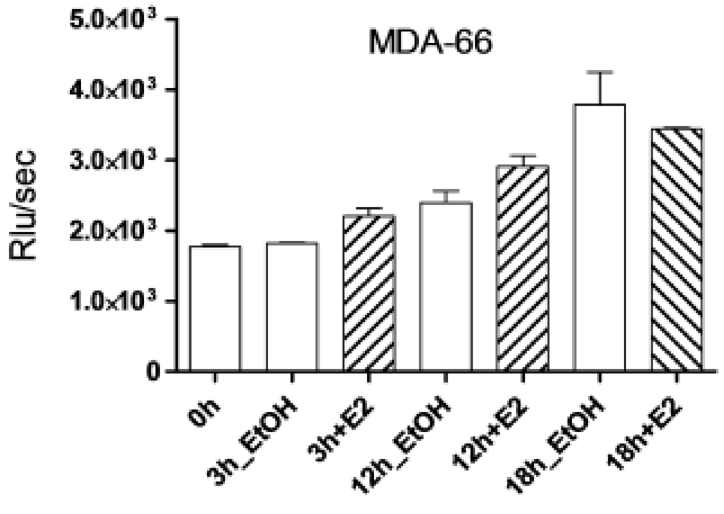
MDA-66
Rlu/sec
5.0x10³
4.0x10³
3.0x10³
2.0x10³
1.0x10³
0
0h
3h_EtOH
3h+E2
12h_EtOH
12h+E2
18h_EtOH
18h+E2

C.

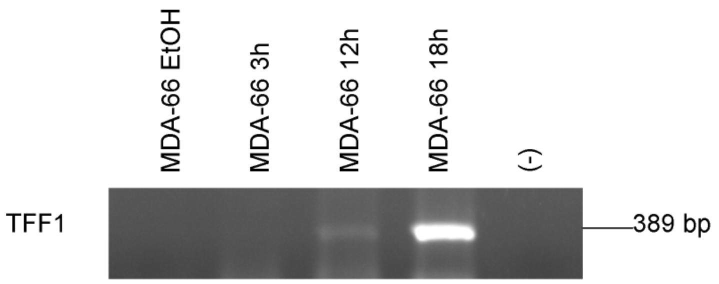

Supplementary Figure 9. A. Relative quantification of *CDC6* 3' UTR short and long isoforms in MDA-66 cells (ER transfected MDA-MB-231) (19). Cells were treated with E2 as described in the Methods section. E2 is known not to have a growth proliferative effect on ER transfected MDA-MB-231 cells (34-37). **B.** E2 treatment did not induce increased BrdU incorporation. Asynchronous MDA-66 cells were grown in phenol red-free media supplemented with 10% dextran-coated-charcoal stripped FBS for 48 hours. Then, cells were treated with BrdU (10 nM) and E2 (10 nM) for the indicated time points along with the ethanol treated controls (EtOH). Incorporated BrdU was detected using the Turner Biosystems Luminometer. Values are given as relative light units (RLU) per sec at respective time points. **C.** *TFF1* is an E2 upregulated gene. MDA-66 cells were treated with ethanol or 10 nM E2 for indicated time points and RNA was isolated. Following DNase treatment, cDNA was synthesized for RT-PCR analysis. E2 treatment resulted with the upregulation of *TFF1* expression.

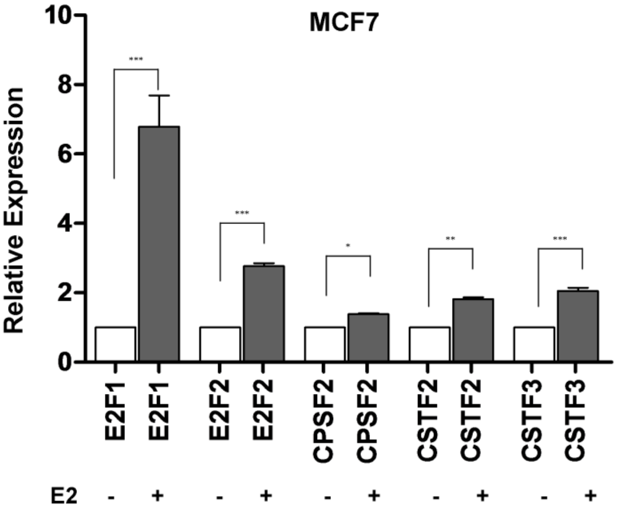

Supplementary Figure 10. *E2F1*, *E2F2* and 3’ UTR processing gene transcripts (*CSTF2* (cleavage stimulation factor, 3' pre-RNA, subunit 2), *CSTF3* (cleavage stimulation factor, 3' pre-RNA, subunit 3) and *CPSF2* (cleavage and polyadenylation specific factor 2) are upregulated in response to E2 treatment in MCF7 cells. Cells were treated with E2 and cDNA was prepared as described in Methods. The fold change for the transcripts was normalized against the reference gene; *SDHA.* Quantification was done using the reaction efficiency correction and ΔΔCq method. The baseline for each transcripts’ in untreated samples were set to 1. Experiment was repeated two independent times with 3 replicas. One-way ANOVA with Tukey’s multiple comparison post test was performed using GraphPad Prism (California, USA). * ($p \leq 0.05$), ** ($p \leq 0.01$), and *** ($p \leq 0.001$) indicates statistical significance.
